# The Needle in the Haystack—Searching for Genetic and Epigenetic Differences in Monozygotic Twins Discordant for Tetralogy of Fallot

**DOI:** 10.3390/jcdd7040055

**Published:** 2020-12-02

**Authors:** Marcel Grunert, Sandra Appelt, Paul Grossfeld, Silke R. Sperling

**Affiliations:** 1 Cardiovascular Genetics, Charité—Universitätsmedizin Berlin, 13125 Berlin, Germany; marcel.grunert@charite.de (M.G.); sandra.appelt@charite.de (S.A.); 2 Berlin Institute of Health (BIH), 10178 Berlin, Germany; 3 DZHK (German Centre for Cardiovascular Research), Partner Site Berlin, 10178 Berlin, Germany; 4 Division of Cardiology, University of California San Diego, Rady’s Hospital MC 5004, San Diego, CA 92123, USA; pgrossfeld@health.ucsd.edu; 5 Department of Biology, Chemistry, and Pharmacy, Freie Universität Berlin, 14195 Berlin, Germany

**Keywords:** congenital heart disease, Tetralogy of Fallot, monozygotic twins, discordant phenotype, genetics, variations, epigenetics, DNA methylation, candidate genes

## Abstract

Congenital heart defects (CHDs) are the most common birth defect in human with an incidence of almost 1% of all live births. Most cases have a multifactorial origin with both genetics and the environment playing a role in its development and progression. Adding an epigenetic component to this aspect is exemplified by monozygotic twins which share the same genetic background but have a different disease status. As a result, the interplay between the genetic, epigenetic and the environmental conditions might contribute to the etiology and phenotype. To date, the underlying causes of the majority of CHDs remain poorly understood. In this study, we performed genome-wide high-throughput sequencing to examine the genetic, structural genomic and epigenetic differences of two identical twin pairs discordant for Tetralogy of Fallot (TOF), representing the most common cyanotic form of CHDs. Our results show the almost identical genetic and structural genomic identity of the twins. In contrast, several epigenetic alterations could be observed given by DNA methylation changes in regulatory regions of known cardiac-relevant genes. Overall, this study provides first insights into the impact of genetic and especially epigenetic factors underlying monozygotic twins discordant for CHD like TOF.

## 1. Introduction

The heart is the first functional organ during embryogenesis, and congenital heart disease (CHD) represents the most common birth defect in humans, affecting about 1% of all newborns [1]. Despite improvements in recognition and therapeutic opportunities based on anatomical, physiological and surgical considerations, CHD remains a leading cause of infant and child mortality. The majority of CHDs are most likely caused be the interplay of multiple subtle genetic, structural genomic or epigenetic alterations [2,3]. In addition, most of them are modulated by gen–environment interactions [2], of which epigenetic alterations are proposed to represent an important transmitter. The symptoms of CHD may vary from none to severe, which attests to its complex nature. This spread is well illustrated in Tetralogy of Fallot (TOF), the most common cyanotic form of CHDs. TOF is characterized by a ventricular septal defect (VSD) with an overriding aorta as well as an obstruction of the right ventricular outflow tract (pulmonary stenosis) and right ventricular hypertrophy. These four anatomical features can all exhibit variable levels of severity.

Over recent decades and in particular with the advent of high-throughput DNA sequencing, several CHD-associated sequence variations have been identified by genetic studies of affected individuals and families [3,4,5,6,7,8]. Using a homogeneous TOF cohort with well-defined features, we recently identified a multigenic background of rare deleterious mutations in several genes, which discriminate TOF cases from controls and which are essential for apoptosis and cell growth, the assembly of the sarcomere as well as for the neural crest and secondary heart field [9]. One central mechanism of epigenetic control is DNA methylation. Thus, we also performed a genome-wide DNA methylation study on myocardial biopsies of TOF and VSD patients [10]. We found clear methylation differences between patients and controls and moreover, between patient groups. However, the underlying specific causes for the majority of CHDs including complex forms such as TOF remain poorly understood.

Monozygotic twins are also known as identical twins because they share nearly 100% of their genetic information stored in DNA. However, they are often discordant for complex diseases such as diabetes, neurodevelopmental disorders, cancer as well as CHD [11,12,13,14]. Considering that monozygotic twins are genetically identical, epigenetic mechanisms like DNA methylation might be modulators of the phenotypic discordance by mediating between the environment and phenotypic expression [13]. For CHD for example, Lyu et al. showed, in the case of an identical twin pair discordant for double outlet right ventricle, a high correlation between hypermethylated promoters based on reduced representation bisulfite sequencing and down-regulated gene expression levels in the patient compared to the healthy twin [14]. In this study, we will investigate for the first-time genome-wide genetic and epigenetic differences between two monozygotic twin pairs discordant for TOF, providing a deeper understanding of the interplay between genetic, structural genomic and epigenetic alterations involved in the etiology of this complex disease.

## 2. Materials and Methods

### 2.1. Study Participants and Ethics Statement

**Figure 1:**
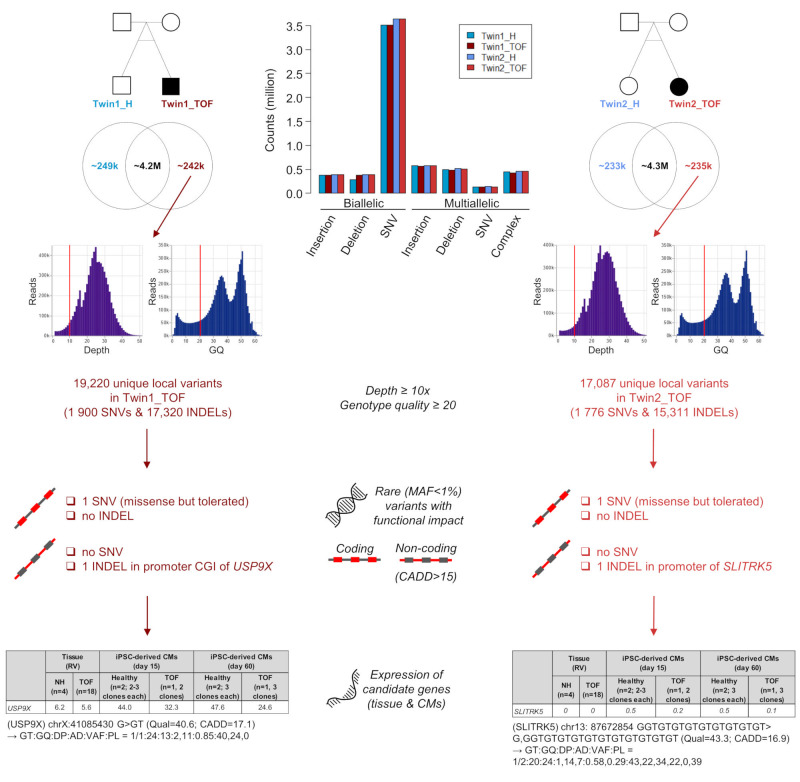
Filtering of local variations and candidate genes identified in affected Tetralogy of Fallot (TOF) twins using whole genome sequencing. Variant positions are based human reference genome (GRCh38.p13/hg38). AD, allelic depth; CADD, combined annotation dependent depletion; CMs, cardiomyocytes; DP, read depth; GT, genotype; GQ, genotype quality; H, healthy; INDEL, insertion and deletion; MAF, minor allele frequency; NH, normal heart; PL = Phred-scaled genotype likelihoods; Qual, base quality (Phred score); RV, right ventricle; SNV, single nucleotide variations; TOF, Tetralogy of Fallot; VAF, variant allele frequency.

To obtain genomic DNA (gDNA) for whole genome sequencing (WGS) and whole genome bisulfite sequencing (WGBS), blood samples were taken from four individuals (two identical twins discordant for TOF, i.e., one affected case each (i.e., Twin1_TOF and Twin2_TOF) and one healthy monozygotic sibling each (i.e., Twin1_H and Twin2_H)). The first pair of twins are male individuals (i.e., Twin1) and the second pair are female individuals (i.e., Twin2) (Figure 1). Since a healthy co-twin in a discordant twin pair serves as a well-matched control with the same genetic background, no further controls were included in this study. The local institutional review board of University of California San Diego approved the study (UCSD IRB protocol #111523) and informed consent was obtained from all participants or guardians. The study protocol conforms to the ethical guidelines of the 1975 Declaration of Helsinki.

### 2.2. Whole Genome Sequencing (WGS)

Genomic DNA from blood of TOF patients and healthy siblings was extracted using Puregene DNA purification kit (Gentra). DNA degradation and contamination were monitored on 1% agarose gels. Moreover, DNA concentration was measured using Qubit DNA Assay Kit in Qubit 2.0 Flurometer (Life Technologies, Carlsbad, CA, USA). Library preparation and sequencing (2 × 150 bp paired-end Illumina sequencing; NextSeq PE150 with Q30 ≥ 80%) was performed by Novogene. Briefly, a total amount of 1 µg DNA per sample was used as input material for the DNA sample preparations. Sequencing libraries were generated using NEBNext DNA Library Prep Kit following manufacturer’s recommendations and indices were added to each sample. The genomic DNA was randomly fragmented to a size of 350 bp by shearing, then DNA fragments were end polished, A-tailed, and ligated with the NEBNext adapter for Illumina sequencing, and further PCR enriched by P5 and indexed P7 oligos. The PCR products were purified (AMPure XP system) and resulting libraries were analyzed for size distribution by Agilent 2100 Bioanalyzer and quantified using real-time PCR.

Each sample was sequenced on four sequencing lanes (Table S1). On average, sequencing resulted in approximately 332 million read pairs per sample (Table S1). The quality of the sequencing samples was checked using FASTQC v1.7 [15] and Qualimap v2.2.1 [16]. All samples passed sequence quality. On average, a duplication rate of 10.4%, a GC content of 40.4% and mean base quality of 37.9 (Phred score) were observed over all samples (Table S1 and Figure S1A). After initial quality check, the reads were mapped to the human reference genome (GRCh38.p13/hg38) using Bowtie2 v2.4.1 with the “very-sensitive” parameter setting, which is more sensitive and more accurate [17]. On average, 98.8% of the reads per sample could be mapped. The average sequencing read depth over the human reference genome is 30.1× with a coverage of 99.5% (Table S2 and Figure S1B). Duplicate reads were marked using Picard tools v1.140 (http://broadinstitute.github.io/picard, v1.140).

Calling of local variations (single nucleotide variations (SNVs) as well as insertions and deletions; INDELs) was performed using DeepVariant v0.10.0 with default parameters [18]. Variants that passed DeepVariant default quality filters and with a genotype quality greater than or equal to 20 and a read depth greater than or equal to 10 were annotated using Ensembl Variant Effect Predictor (VEP; release 100) [19]. Local variations with a minor allele frequency greater or equal than 0.01 (1%) in the 1000 Genomes or Genome Aggregation Database (gnomAD) global populations [20] as well as variations with low functional impact based on Ensembl calculated variant consequences were filtered out [19]. Non-synonymous variants must be predicted to be damaging by either PolyPhen-2 [21], SIFT [22] or MutationTaster2 [23]. Moreover, variants in the non-coding region were filtered for those with a CADD (Combined Annotation Dependent Depletion) score greater than 15 (Phred-scaled) [24], a cutoff on deleteriousness. Structural variations (SVs) were called using Manta v1.6.0 with default parameters [25]. Moreover, common SVs based on gnomAD-SV were filtered out [20]. Copy number variations (CNVs) were called using Control-FREEC v11.5 with default parameters [26]. Both SVs and CNVs were further overlapped with genomic features obtained from VEP.

### 2.3. Whole Genome Bisulfite Sequencing (WGBS)

For WGBS, gDNA obtained from blood of the two twin pairs (in total four individuals) was extracted using Puregene DNA purification kit (Gentra) and further used to create bisulfite-treated DNA libraries. Library preparation and sequencing was performed by Novogene using EZ DNA Methylation Gold Kit from Zymo Research and 150 bp paired-end Illumina-Kit. Briefly, unmethylated cl857 Sam7 Lambda DNA (48,502 bp, Promega D1521) was combined with gDNA to act as an internal control to monitor the bisulfite conversion rate. Afterwards, DNA was sheared into 200–400 bp fragments, sequence ends were repaired and 3′ ends were adenylated. Next, methylation sequencing adapters were added and DNA was treated with bisulfite. Treatment of DNA with sodium bisulfite deaminates unmethylated cytosines to uracil while methylated cytosines are resistant to this conversion, allowing therefore for the discrimination between methylated and unmethylated CpG sites. Finally, bisulfite treated library was subjected to PCR amplification followed by standard DNA paired-end Illumina sequencing (HiSeq PE150 with Q30 ≥ 80%). In general, sodium bisulfite pre-treatment of DNA coupled with high-throughput sequencing allows us to study DNA methylation quantitatively and genome-wide at single cytosine site resolution.

Each sample was sequenced on six sequencing lanes (Table S3). On average, sequencing resulted in approximately 344 million read pairs per sample (Table S3). The quality of the sequencing samples was checked using FASTQC v1.7 and Qualimap v2.2.1. All samples passed sequence quality. On average, a duplication rate of 10%, a GC content of 21.1% and mean base quality of 36.95 (Phred score) were observed over all samples (Table S3 and Figure S2). The bisulfite conversation was efficient based on the base composition (<1–2% of C in the forward strand and G in the reverse strand) (Figure S3A) and based on the bisulfite conversion rate (>99.7%) (Figure S3B). The bisulfite conversion efficiency was calculated for each sample based on unmethylated control sequences (spike-ins) added to the library prior to fragmentation (i.e., 100—percentage of C methylated in CpG context when mapping against the control genome *Enterobacteria phage lambda*). After initial quality assessment, adapter trimming was performed by Trim Galore v0.4.4 [27]. Afterwards, sequencing reads were mapped to the human reference genome (GRCh38.p3) using Bismark v0.18.2 [28]. On average, 75% of the ~344 million reads per sample could be mapped (Table S4). After mapping, reads of all sequencing lanes were merged for each sample using SAMtools v1.2 [29] and subjected to deduplication using Bismark. Out of ~258 million mapped reads per sample, ~229 million left after deduplication (Tables S4 and S5). All mapped reads have a high mean base quality of ~35.7 (Phred score) with a GC content of ~21.3% (Figure S4A and Table S5). The average sequencing depth is 21.1× per base (Figure S4B and Table S5). To correct for methylation bias at 3′ and 5′ end of each read, MethylDackel v0.3.0 was used [30]. For forward reads, no cutoffs were suggested; however, there are clear drops in the methylation level at the beginning (15–18 bp) and end (149 bp) of the reads for which cutoffs were suggested by MethylDackel (Figure S5A) and incorporated during methylation level extraction using Bismark. As expected, the samples have, on average, a CpG methylation rate of 82.5%. CHG and CHH methylation rates are very low at under 1% each (Figure S5B and Table S5). Differential methylation analyses of CpG sites between two samples are performed using methylSig v0.4.4 [31]. As methylSig only allows comparisons with at least two samples per group, each sample was duplicated. Furthermore, CpGs were filtered by coverage with a minimum coverage of 10 and a maximum of 500 (default values). Differential methylation is defined by a methylation difference of at least 25%. Differential methylated CpGs are further overlapped (at least 1 bp) or associated (i.e., nearest gene approach) with hg38 annotated genomic features as described in detail elsewhere [10]. Briefly, the overlap includes 50,497 promoters of UCSC RefSeq genes (−500 bp/+2 kb to TSS); 30,477 CpG islands (CGIs) based on UCSC track “cpgIslandsExt”; promoter CGIs (i.e., overlap of promoter and CGIs with at least 1 bp), CGI shores (i.e., regions outside CpG islands but within 2 kb of any CpG island), transcription factor binding sites (TFBS) predicted by the Transfac Matrix Database and conserved in the multiple alignment of human, mouse and rat, including cardiac transcription factors (TFs); and cardiac enhancers (p300 ChIP-seq data of human adult and fetal hearts [32]).

### 2.4. Filtering for Disease-Relevant Genetic and Epigenetic Alterations

Candidate genes with genetic, structural genomic or epigenetic alterations were overlapped with various datasets to filter for known or possible disease-relevant genes. This includes heart- and muscle relevant genes (865 genes based on several resources) [9]; cardiovascular-associated genes (list of 4275 genes created by the Cardiovascular Gene Annotation Initiative in collaboration with EMBL-EBI); genes which are differentially expressed in CHD patients (in particular TOF, VSD, atrial septal defect (ASD), hypoplastic left heart syndrome (HLHS), transposition of the great arteries combined with pulmonary artery (TGA-PS)) [9,33,34,35,36,37]; genes which are differentially methylated in the promoter, gene body or related enhancer of CHD patients (in particular TOF, VSD, LS-CHD) [10,38,39,40,41]; genes which overlap CNVs associated with CHD (including TOF, LS-CHD, HLHS and conotruncal defects) [42,43,44,45,46,47,48,49,50,51,52,53,54,55,56]; known human CHD genes [3,6,54]; and genes which are differentially expressed and targeted by differentially expressed microRNAs in CHD patients (TOF and HLHS) [57,58,59]. Moreover, candidate genes must be expressed (RPKM (reads per kilo base per million mapped reads) or TPM (transcript per million) value > 1) in the right ventricular tissue of normal or TOF hearts [9], or during cardiac differentiation (i.e., in cardiomyocytes of day 15 and/or day 60 derived from induced pluripotent stem cells of healthy, unaffected individuals and TOF patients) [60].

### 2.5. Statistics

General bioinformatics and statistical analyses were conducted using R (including Bioconductor packages) and Perl.

## 3. Results

### 3.1. Genomic Variations in Affected Twins

Whole genome sequencing of monozygotic twins resulted in approximately 4.4–4.6 million local variations (i.e., SNVs and INDELs) for each twin, with SNVs constituting the largest class of called genomic variations. Out of these raw variants, up to 6% are unique for either twin. Considering that monozygotic twins are genetically identical, this number seems very high; however, there are differences in terms of sequencing depth and quality, which may result in different calling results. Using a minimum sequencing depth of 10× and a genotype quality of at least 20, the number of local variants drops in Twin1_TOF from 4,167,698 to 19,220 (0.46%) and in Twin2_TOF from 4,331,595 to 17,087 (0.39%) unique ones compared to the respective unaffected twin (Figure 1). To identify possible disease-associated alterations, these unique variants were further annotated and filtered for rare variants (MAF < 1%) with functional impact on the coding or non-coding genomic sequence. For the coding part, this resulted in no single INDEL and only one missense SNV for each affected twin, which are further predicted to be tolerated (Figure 1). For the non-coding sequence of Twin1_TOF, there is a homozygous INDEL (i.e., insertion) in the promoter region of *Ubiquitin Specific Peptidase 9 X-Linked* (*USP9X)*. The promoter also represents a CpG island (CGI). In Twin2_TOF, we also identified no SNV but one INDEL in the promoter of *SLIT and NTRK Like Family Member 5* (*SLITRK5)*. Both non-coding variants are potentially pathogenic with a CADD score greater than 15. The related genes were further checked for being expressed in the human heart. Moreover, considering that TOF is a developmental disorder, these genes should be expressed during development. Thus, we examined their expression in the right ventricular tissue of normal and TOF hearts [9] as well as during cardiac differentiation using cardiomyocytes derived from induced pluripotent stem cells of healthy, unaffected individuals and TOF patients [60]. Out of these two genes, *SLITRK5* is not expressed in the heart or during cardiomyocyte differentiation (Figure 1). We further overlapped the two genes with known cardiovascular-associated genes and several CHD-related datasets, but without any overlap for both genes. In addition to unique variants in the two affected TOF twins (i.e., variants that do not occur in the healthy sibling), we also searched for variations with zygosity differences between the healthy and affected siblings. However, after filtering there is no SNV or INDEL in the coding and non-coding sequence of both twin pairs, which can explain the phenotype differences (Figure S6).

### 3.2. Structural Genomic Variations in Affected Twins

Besides local variations, we are interested in copy number variations as well as structural variations, which are unique for the affected TOF twins. For Twin1_TOF, we identified 11 CNVs, which are associated with 9 genes (Table S6). The majority of these CNVs are copy number gains (i.e., 9 out of 11) and only two represent a copy number loss. For Twin2_TOF, we found four CNVs (two gains and two losses), of which only one copy number gain affects a genomic feature, namely the rRNA *RNA5SP19*. The very few genomic features affected by CNVs in Twin1_TOF are either protein-coding or T cell receptors, where the latter represent an adaptive immune response (Table S6). The protein-coding genes were checked for cardiac expression and further overlapped with a list of cardiovascular-associated genes and other CHD-related datasets. The latter revealed only for *UDP Glucuronosyltransferase Family 2 Member B17* (*UGT2B17)* an overlap with cardiovascular-associated genes and known CNVs in CHD patients; however, the gene is not expressed in the normal or TOF heart or during cardiomyocyte differentiation. The only expressed gene associated with a CNV in twin1_TOF is *Fc Fragment of IgA Receptor* (*FCAR)*, but without any overlap to other cardiac-related annotations or datasets. In summary, there is no real candidate gene with a CNV in both affected twins.

To find possible disease-relevant SVs, we filtered for unique ones in the affected twins, which are rare and have a high or modifier functional impact. In total, we found 30 rare SVs in Twin1_TOF and 20 rare SVs in Twin2_TOF with modifier impact only, affecting 20 and 13 genes, respectively (Tables S7 and S8 and Figure S7). Both affected twins share five SVs. Overall, 83% of the genes associated with SVs are expressed in the normal or TOF heart or during cardiac differentiation. Moreover, the majority of these expressed genes overlap with several cardiac related genes or datasets. For example, *ANTXR Cell Adhesion Molecule 1 (ANTXR1)* and *L3MBTL Histone Methyl-Lysine Binding Protein 4* (*L3MBTL4)* with SVs in Twin1_TOF are also differentially expressed (significantly up-regulated) in right ventricle of TOF patients compared to normal hearts [9]. Moreover, both are differentially methylated in VSD patients compared to normal hearts (i.e., significantly hypermethylated cardiac enhancer near *ANTXR1* and hypermethylated gene body of *L3MBTL4*) [10]. The genes with SVs in Twin2_TOF, encoding for *Bardet-Biedl Syndrome 2* (*BBS2*) and *Teneurin Transmembrane Protein 4* (*TENM4*), are also associated with differential DNA methylation in VSD cases versus normal hearts (i.e., significantly hypermethylated gene body of *BBS2* and hypermethylated promoter of *TENM4*) [10].

### 3.3. DNA Methylation Differences between Discordant Twins

**Figure 2:**
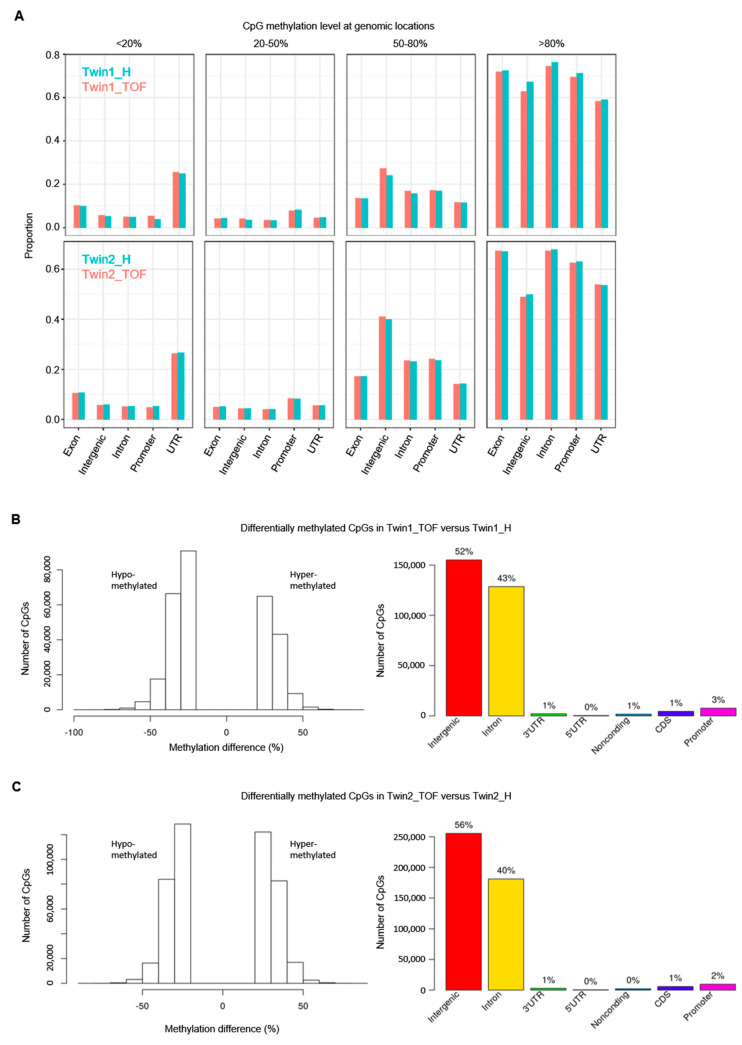
DNA methylation levels and differences of CpG sites in twins and their genomic locations. (**A**) Overview of methylation levels of CpGs at genomic locations. Proportions of CpG are given over the total count for each feature (no pairwise comparison of single CpG sites). (**B**) Differentially methylated (hypo- and hyper-methylated) CpG sites in Twin1_TOF compared to Twin1_H and their genomic locations. (**C**) Differentially methylated (hypo- and hyper-methylated) CpG sites in Twin2_TOF compared to Twin2_H and their genomic locations. Differentially methylated CpGs with at least 25% methylation difference between affected (*_TOF) and healthy (*_H) twin.

To investigate the impact of DNA methylation changes between discordant twins, we performed WGBS and studied the methylation level of approximately 28 million CpG sites in the human genome. In general, the global CpG methylation shows no obvious differences between each affected and healthy twin (Figures S8 and S9A), with a somewhat higher coverage in Twin1_TOF compared to Twin1_H and a similar one for Twin2_TOF and Twin2_H (Figure S9B). Moreover, genomic features associated to genes have a higher methylation level (Figure 2A).

Next, we searched for differentially methylated CpG sites (DMCs) with at least 25% differences between the discordant twins. We found 299,643 DMCs in Twin1 and ~150,000 more DMCs (in total 457,108) in Twin2 (Table 1). For Twin1, there are more hypomethylated CpGs, while Twin2 is more balanced regarding hypo- and hyper-methylation (Figure 2B,C, left each). However, the overlap of DMCs with genomic features is similar for both twin pairs, with the majority of DMCs in non-coding regions (intergenic and intronic; Figure 2B,C, right each). Nevertheless, there are DMCs in coding and even more interestingly in regulatory regions such as promoters, CGIs, promoter CGIs, CGI shores, TFBS (cardiac and/or located in promoters) and cardiac enhancers (Table 1).

**Figure 3:**
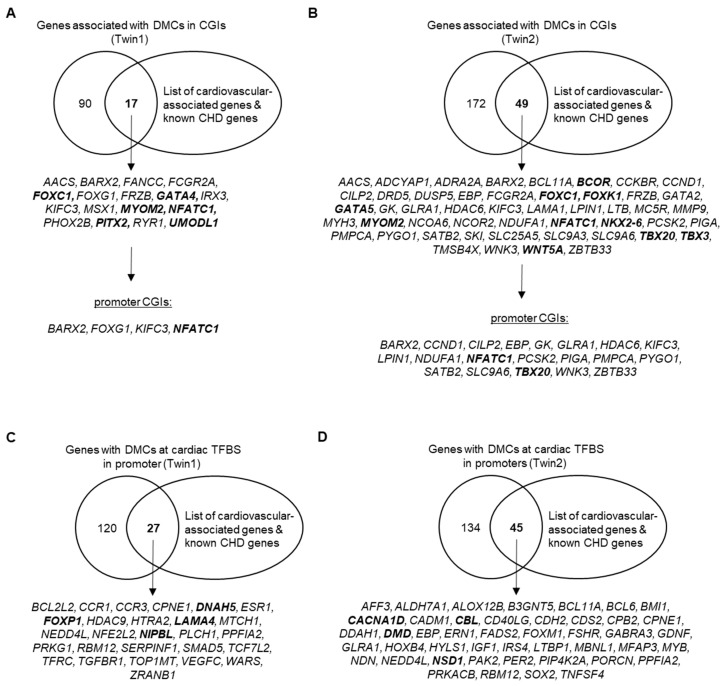
Overlap of protein-coding genes associated with differentially methylated CpGs in CGIs and promoter CGIs or at cardiac TFBS in promoters with cardiovascular-associated genes and known CHD genes. Known CHD genes are marked in bold. (**A**) Genes associated with DMCs of Twin1_TOF versus Twin1_H (i.e., Twin1) in CGIs or promoter CGIs. (**B**) Genes associated with DMCs of Twin2_TOF versus Twin2_H (i.e., Twin2) in CGIs or promoter CGIs. (**C**) Genes with DMCs of Twin1 at cardiac TFBS in their promoter. (**D**) Genes with DMCs of Twin2 at cardiac TFBS in their promoter. CGIs; CpG islands; CHD, congenital heart disease; DMCs, differentially methylated CpG sites; TFBS, transcription factor binding sites.

Of particular interest is the methylation pattern of dense regions of CpGs, the CGIs. For Twin1, we found 397 DMCs in CGIs and 131 of these are located in promoters. For Twin2, there are 713 DMCs in CGIs and the majority (in total 429) overlap promoter regions. Next, we overlapped (promoter) CGI-associated genes with cardiovascular-associated genes including known CHD genes and found several cardiac-related genes (Figure 3A,B). In total, there are 4 and 20 genes with DMCs in promoter CGIs for Twin1 and Twin2, respectively. Interestingly, 3 out 4 genes in Twin1 also harbors DMCs in Twin2. These genes are *BARX Homeobox 2* (*BARX2*), *Kinesin Family Member C3* (*KIFC3*) and *Nuclear Factor of Activated T Cells 1* (*NFATC1*). The latter is a transcription factor required for valve formation [61] and thus, a known CHD gene. In addition to *NFACT1*, there is another transcription factor and well-known CHD gene in Twin2 with DMCs in its promoter CGI, namely *T-Box Transcription Factor 20* (*TBX20*) [62,63].

Besides the regulatory impact of CpG Islands, we also examined genes that have DMCs at TBFS in their promoter. In total, 27 and 45 genes in Twin1 and Twin2, respectively, harbor such DMCs and overlap with cardiovascular-associated genes (Figure 3C,D). Four of these genes were found between both twin pairs (i.e., *CPNE1, NEDD4L, PPFIA2, RBM12*). Moreover, there are four different known CHD genes with DMCs at TBFS in their promoter in Twin1 (i.e., *DNAH5, FOXP1, LAMA4, NIPBL*) and Twin2 (i.e., *CACNA1D, CBL, DMD, NSD1*).

**Figure 4:**
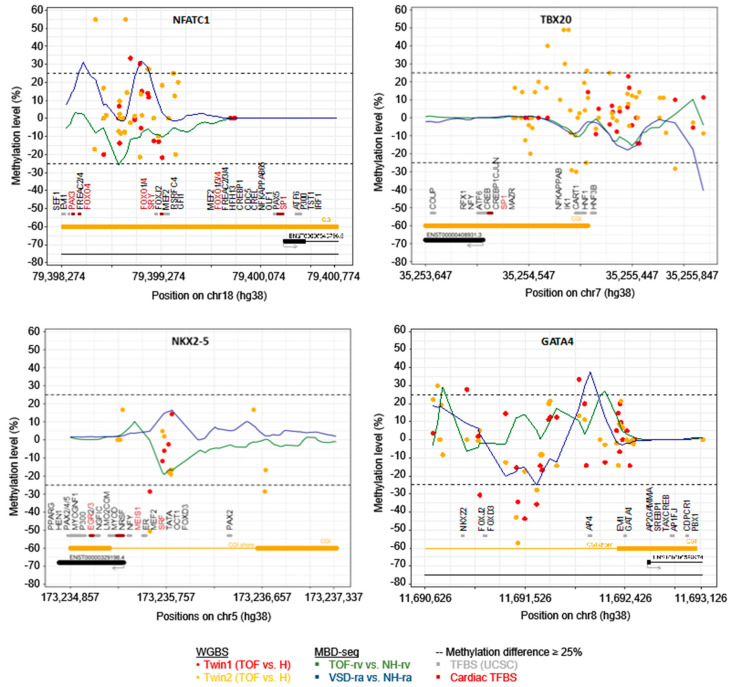
DNA methylation level at promoter regions of *NFATC1, TBX20, NKX2-5* and *GATA4* in healthy and affected twins obtained by WGBS (*n* = 1 each; coverage at CpGs ≥ 5×) as well as in right ventricular or atrial tissue of normal hearts and patients with TOF or VSD obtained by methyl-CpG binding domain protein-enriched genome sequencing (MBD-seq) (*n* = 7 for TOF-rv; *n* = 8 for VSD-ra; *n* = 3 for NH-rv; *n* = 4 for NH-ra). Promoters are defined as −2 kb/+500 bp of transcriptional start site. CGIs and CGI shores are indicated by orange line at the bottom. Moreover, binding sites of transcription factors (obtained from UCSC) are provided in gray or for cardiac factors in red. For WGBS, methylation at single CpG is provided. CGIs, CpG islands; NH, normal heart; H, healthy; RA, right atrium; RV, right ventricle; TFBS, transcription factor binding site; TOF, Tetralogy of Fallot; VSD, ventricular septal defect; WGBS, whole genome bisulfite sequencing.

Figure 4 shows the difference in the methylation level of both twins at the promoter of four selected known CHD genes, including TFBS in these regions. In addition, the mean methylation level obtained from methyl-CpG binding domain (MBD) protein-enriched genome sequencing (MBD-seq) of tissues from normal hearts as well as from a homogenous group of TOF and VSD patients is provided [10]. Figure 4 above shows the methylation level at the promoter CGI of *NFATC1* and *TBX20*. The methylation level at the promoter of two essential cardiac transcription factors, *NKX2-5* and *GATA4*, which both harbor single DMCs in their promoter region with more than 25% methylation difference between healthy and affected twin, is shown in the bottom of Figure 4. In general, the observations in WGBS partially differ with the methylation pattern observed in MBD-seq, which may be due to the resolution (1 bp in WGBS vs. ~150 bp in MDB-seq) and coverage (i.e., single CpGs in WGBS ≥ 5×) or simply based on the homogeneity of the affected twin compared to other TOF and VSD patients. However, for both WGBS and MBD-seq one can observe methylation alterations in the promoter of these selected genes.

## 4. Discussion

Out of millions of unique local variations (i.e., SNVs and INDELs) for each affected twin obtained from WGS, we identified no single candidate gene in one of the two monozygotic discordant twin pairs, which might explain the difference regarding TOF (Figure 1). Searching for candidate genes affected by structural genomic variations, we observed in both TOF twins several SV candidates but no relevant CNVs. For SVs, there are several candidates in both affected twins who are also expressed in the adult heart and during cardiac differentiation (Table S9). Moreover, most of these expressed genes also overlap with cardiovascular-associated genes and CHD-related datasets. Most interestingly for Twin1_TOF, the affected genes *ANTXR* and *L3MBTL* are also found to be differentially expressed in right ventricle of TOF versus normal hearts [9] and in addition, differentially methylated in right atrium of VSD versus normal hearts [10]. *L3MBTL* is involved in transcriptional repression [64]. Its MBT domain binds to methylated histone residues, which is linked to the formation and maintenance of heterochromatin [65]. For Twin2_TOF, the affected genes *BBS2* and *TENM4* are also differentially methylated in VSD versus normal hearts [10]. However, none of the affected genes is known to be involved in TOF or CHD in general so far. Moreover, since we do not have any tissue available from the twins (only blood samples), we unfortunately cannot make any statement regarding the regulatory impact of these SVs on the expression of related genes. Due to ethical restrictions, it is further practically impossible to obtain such tissue from the healthy, unaffected twin; however, follow-up studies based on induced pluripotent stem cells and derived cardiomyocytes might be an option to verify the evidence for their causative impact.

As mentioned above, some of the genes with SVs in the TOF twins are already known to be associated with differential DNA methylation in cardiac tissue of patients with TOF and in its sub-feature, VSD [10]. To study the epigenetic differences based on DNA methylation between the monozygotic discordant twin pairs, we used blood sample for WGBS as for WES analysis of genetic and structural genomic alteration. CpG methylation can persist in steady-state from early embryonic development throughout a lifetime, and only a fraction of 22% of autosomal CpGs shows dynamic methylation in the normal developmental context [66,67]. With respect to the study of altered DNA methylation pattern, this enables the analysis of up to 80% of CpG methylations in cells independent from the affected cell type. Overall, we observed no differences based on the global CpG methylation between each of the two twin pairs. However, we found numerous single CpGs differentially methylated with more than 25% difference between the twins (Table 1). Most interesting are those DMCs in promoters that also overlap with CGIs or TFBS (Figure 3). Usually, CGIs are largely unmethylated [68] and frequently located in promoters [69]. Multiple methylated CpGs in promoter CGIs cause stable silencing of genes and thus, hypermethylated promoter CGIs are often known to be disease-related [70]. For both affected twins, we found DMCs in the promoter CGI of a gene encoding for the TF NFATC1 (top left of Figure 4), which is required for normal valve formation [61]. Moreover, genetic and structural genomic alterations in *NFATC1* have already been identified in patients with CHD such as VSD or tricuspid atresia [71,72]. For Twin2_TOF, we identified several DMCs in the promoter CGI of *TBX20* (top right of Figure 4), a cardiac TF [63]. Mutations in *TBX20* have been identified in different types of cardiac defects regarding septation, chamber growth, and valvulogenesis [62]. Tbx20 interacts with the TF Gata4 to active both Mef2c and *Nkx2-5* enhancers [73], which all are core cardiac TFs [63]. These factors regulate each other’s expression, partly with combinatorial impact of downstream targets. Disturbances of their associated cardiac regulatory network have been associated with various congenital heart malformations [63]. For both cardiac TFs GATA4 and *NKX2-5*, we found DMCs in the promoter region of both affected twins, who further represent CGI shores (bottom of Figure 4). These regions are within 2 kb of a CGI and they are known to occur most of the tissue-specific methylation [74].

There are many other candidate genes associated or overlapping DMCs with different regulatory features (Table 1); however, it clearly shows that the difference between the two monozygotic discordant twin pairs is rather observed in epigenetic alterations than on the genetic or structural genomic level. Nevertheless, the impact of these epigenetic variations on candidate gene expression and thus, their causality for the phenotypic discordance needs to be verified by further studies, in functional respect but also in terms of cases and comparisons (i.e., this study comprises just two cases with each one affected vs. one healthy twin comparison). However, our study confirms other studies on identical twins that discordances in a disease such as CHD cannot be explained by genetic or structural genomic differences [14,75,76,77,78,79]. Moreover, blood-derived DNA might be chimeric between the identical twins and as shared blood circulations have been found during embryogenesis in most monozygotic twin pregnancies [80], hematopoietic stem cells can be transferred between twins. Such a created hematopoietic system might mask the underlying genetic, structural genomic and also epigenetic differences between the monozygotic twin discordant for a disease like TOF [75].

Recent large-scale studies with up to thousands of CHD cases and of controls only show possible genetic or structural genomic causes for a minority of cases [4,7,44,53,81]. The reasons for this might be found in the multifactoriality paired with complex inheritance patterns and low prevalence [82]. Moreover, the majority of cases occur sporadic, possibly triggered by stochastic or environmental events during maternal pregnancy [83,84]. The latter is accompanied by epigenetic changes such as those related to histone modifications and DNA methylation, important modulators of gene–environment interactions. Moreover, deregulated microRNA abundance levels also seem to play a role in the development of CHD in twins [85]. Stochastic events such as an unequal division of the inner mass cells during twinning or an unequal allocation of developmental markers and precursor cells might be responsible for a discordance in monozygotic twins [86], which might be of interest with respect to a possible premature stop of cellular growth in TOF. Moreover, this may result, for example, in altered sub-populations of ventricular and atrial cardiomyocytes [87], which in turn might be disease-causing. In general, monozygotic twins are up to seven times more likely to develop a congenital heart malformation, which further usually occurs in just one of the twins [86,88]. Reasons for this increased risk include the twin–twin competition for maternal resources resulting in, for example, unequal placental sharing, unequal sharing of the vascular system or other placental mechanisms like diffusion and endocrine function, which increases the probability of a skewed in utero environment affecting the twins [86,89,90,91]. Interestingly, a study based on the Danish Twin Registry showed (unlike several other studies) that there is no higher risk of CHD in monozygotic twins compared to dizygotic ones [92]. This indicates that the intrauterine environment of twin gestation is predisposed to the development of CHD in a certain way. Moreover, it is suggested that twin gestation changes maternal nutrition to the fetus and nutritional deficiencies might increase the sensitivity of the fetus to other teratogenic factors such as prenatal exposure or poor maternal health [92].

The search for the underlying mechanisms of CHD in twins is like searching for the needle in the haystack. The four pieces of the TOF pathology can all exhibit variable levels of severity and as a result, no two TOF cases are the same. However, studying the genetic and epigenetic mechanisms in identical discordant twins can provide important insights into complex diseases such as congenital heart malformations. Therefore, we studied here the genetic, structural genomic and epigenetic differences of two identical twin pairs discordant for TOF. The two cases show common and unique patterns of epigenetic modifications, which might also be related to a common and unique level of pathological features. Understanding the mechanisms that trigger the molecular plasticity, namely the underlying genetic composition, the epigenetic make-up and environmental insults, is quite challenging but will enhance our current knowledge of CHD.

## Figures and Tables

**Table 1:** Differentially methylated CpG sites with ≥25% methylation difference between the healthy and affected twin and their overlap (≥1 bp) or association (i.e., nearest gene approach) with different genomic features. CGIs, CpG islands; DMCs, differentially methylated CpGs; H, healthy; TFBS, transcription factor binding site; TOF, Tetralogy of Fallot.

	Twin1_TOF vs. Twin1_H	Twin2_TOF vs. Twin2_H
*Differentially methylated CpGs (DMCs) with ≥25% methylation difference*	*299,643*	*457,108*
DMCs in promoters	18,052	22,537
DMCs in CpG islands (CGIs)	397	713
DMCs in promoter CGIs	131	429
DMCs in CGI shores	17,337	21,455
DMCs at TFBS	23,716	31,789
DMCs at cardiac TFBS	2066	2751
DMCs at TFBS in promoters	2125	2929
DMCs at cardiac TFBS in promoters	215	264
DMCs in cardiac enhancers (p300)	2042	2518

## Supplementary Materials

The following are available online at https://www.mdpi.com/2308-3425/7/4/55/s1, Figure S1: Statistics of reads obtained from whole genome sequencing. Figure S2: Statistics of input reads obtained from whole genome bisulfite sequencing. Figure S3: Bisulfite conversion efficiency. Figure S4: Quality measurements of deduplicated mapped reads obtained from whole genome bisulfite sequencing. Figure S5: Methylation bias and rates. Figure S6: Filtering of possible disease-relevant local variations identified in affected TOF twins based on zygosity differences between healthy and affected sibling. Figure S7: Filtering of possible disease-relevant structural variations (SVs) identified in affected TOF twins using whole genome sequencing. Figure S8: Global DNA methylation levels of twins. Line plots show the DNA methylation along the chromosomal length (GRCh38.p13/hg38). Figure S9: DNA methylation rate and coverage of CpGs. Table S1: Overview of sequencing reads obtained from whole genome sequencing. Table S2: Statistics of read mapping, coverage and read depth in each sample obtained from whole genome sequencing. Table S3: Overview of sequencing reads obtained from whole genome bisulfite sequencing. Table S4: Mapping result of reads obtained from whole genome bisulfite sequencing. Table S5: Read statistics after mapping and deduplication as well as methylation rates over CpGs, CHGs and CHHs in each sample obtained from whole genome bisulfite sequencing. Table S6: Copy number variations identified in Twin1_TOF and Twin2_TOF based on whole genome sequencing data. Table S7: Structural variations uniquely identified in Twin1_TOF based on whole genome sequencing data. Table S8: Structural variations uniquely identified in Twin2_TOF based on whole genome sequencing data. Table S9: Candidate genes with structural variations in Twin1_TOF and/or Twin2_TOF.
