# Supplementary material for: The Needle in the Haystack—Searching for Genetic and Epigenetic Differences in Monozygotic Twins Discordant for Tetralogy of Fallot
Source: J Cardiovasc Dev Dis. 2020 Dec 2;7(4):55. doi: 10.3390/jcdd7040055 (PMC7761217; doi:10.3390/jcdd7040055)
Supplement: Supplementary file 1 [file jcdd-07-00055-s001.pdf]

The needle in the haystack - searching for genetic and epigenetic differences in monozygotic twins discordant for Tetralogy of Fallot

Marcel Grunert, Sandra Appelt, Paul Grossfeld & Silke R. Sperling

Supplementary Materials

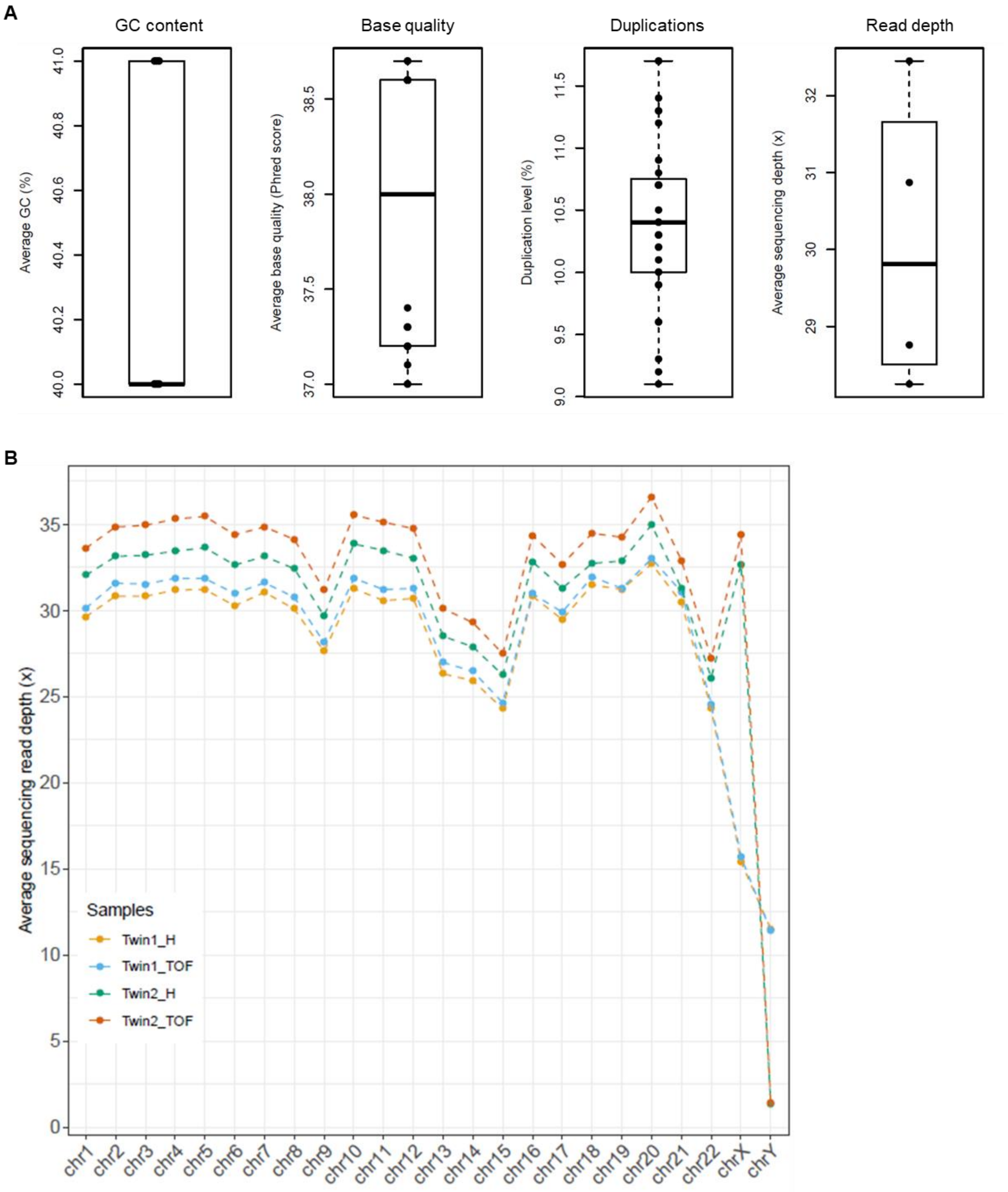

Figure S1. Statistics of reads obtained from whole genome sequencing. (**A**) Ranges of GC content, duplication level and read quality scores of total reads over all samples (n=16, i.e., values for each lane of four samples) as well as mean read depth per base after mapping (n=4). (**B**) Average sequencing read depth across the human reference genome (hg38).

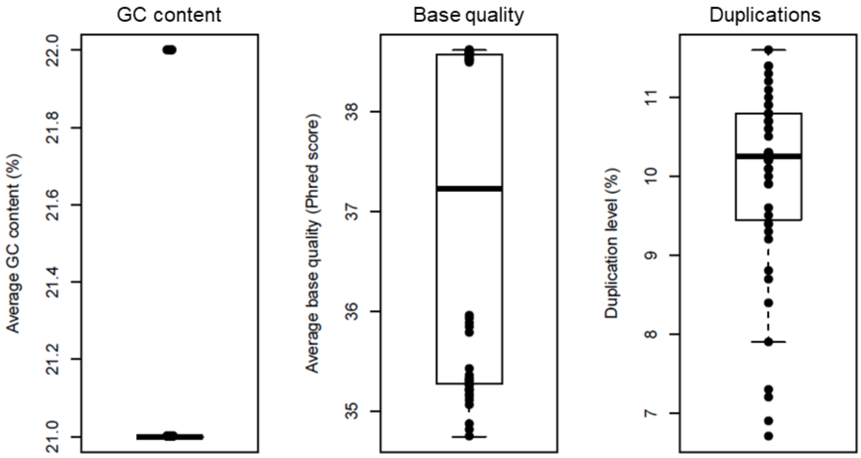

Figure S2. Statistics of input reads obtained from whole genome bisulfite sequencing. Values for each lane of four samples (i.e., n=24).

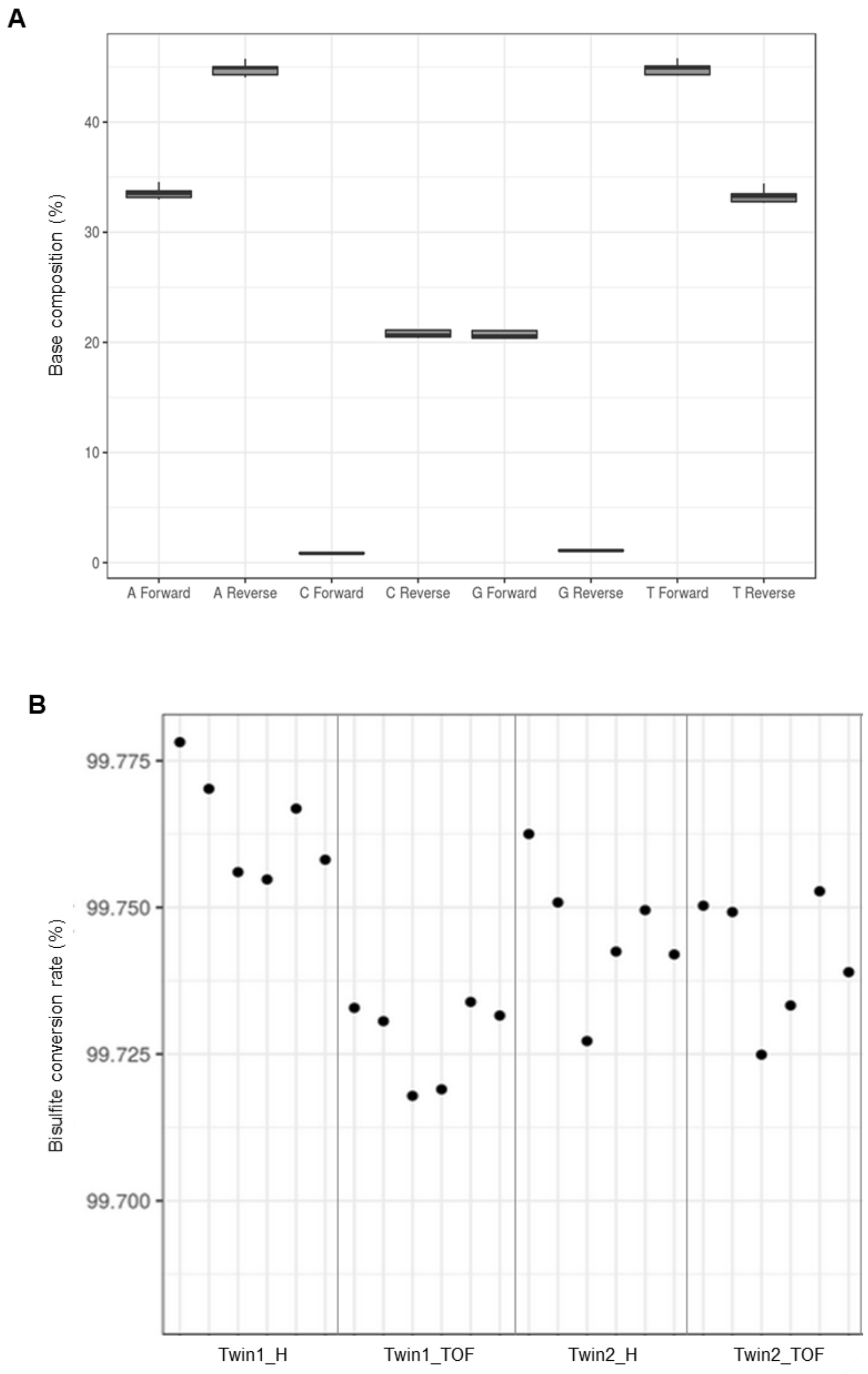

Figure S3. Bisulfite conversion efficiency. (**A**) Base compositions on forward and reverse strand over all samples. (**B**) Bisulfite conversion rate over all samples. Each sample was sequenced on six sequencing lane (n=6).

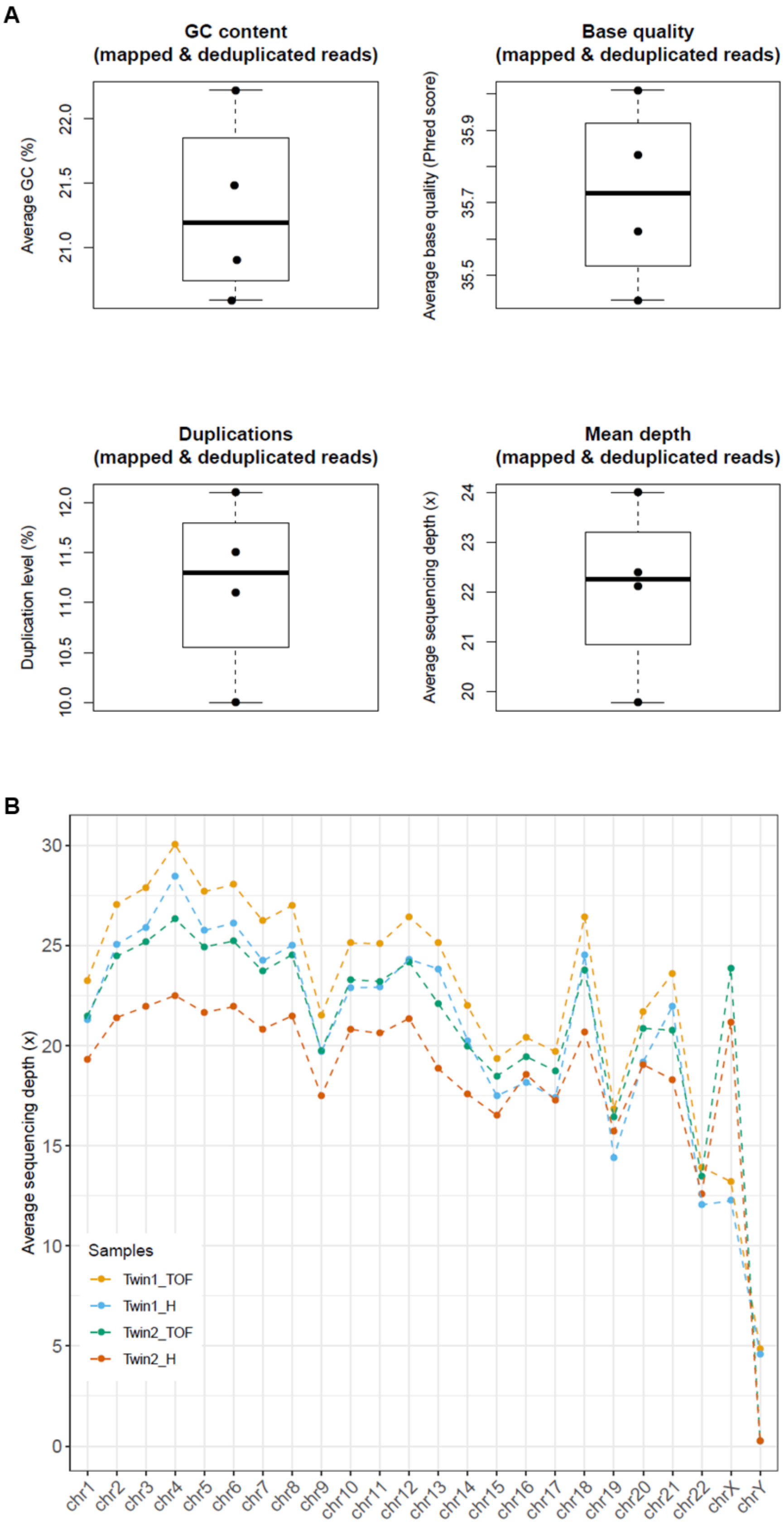

Figure S4. Quality measurements of deduplicated mapped reads obtained from whole genome bisulfite sequencing. (**A**) Ranges of GC content, read quality scores and duplication level of deduplicated mapped reads over all samples as well as mean read depth per base. (**B**) Average sequencing read depth across the human reference genome (hg38).

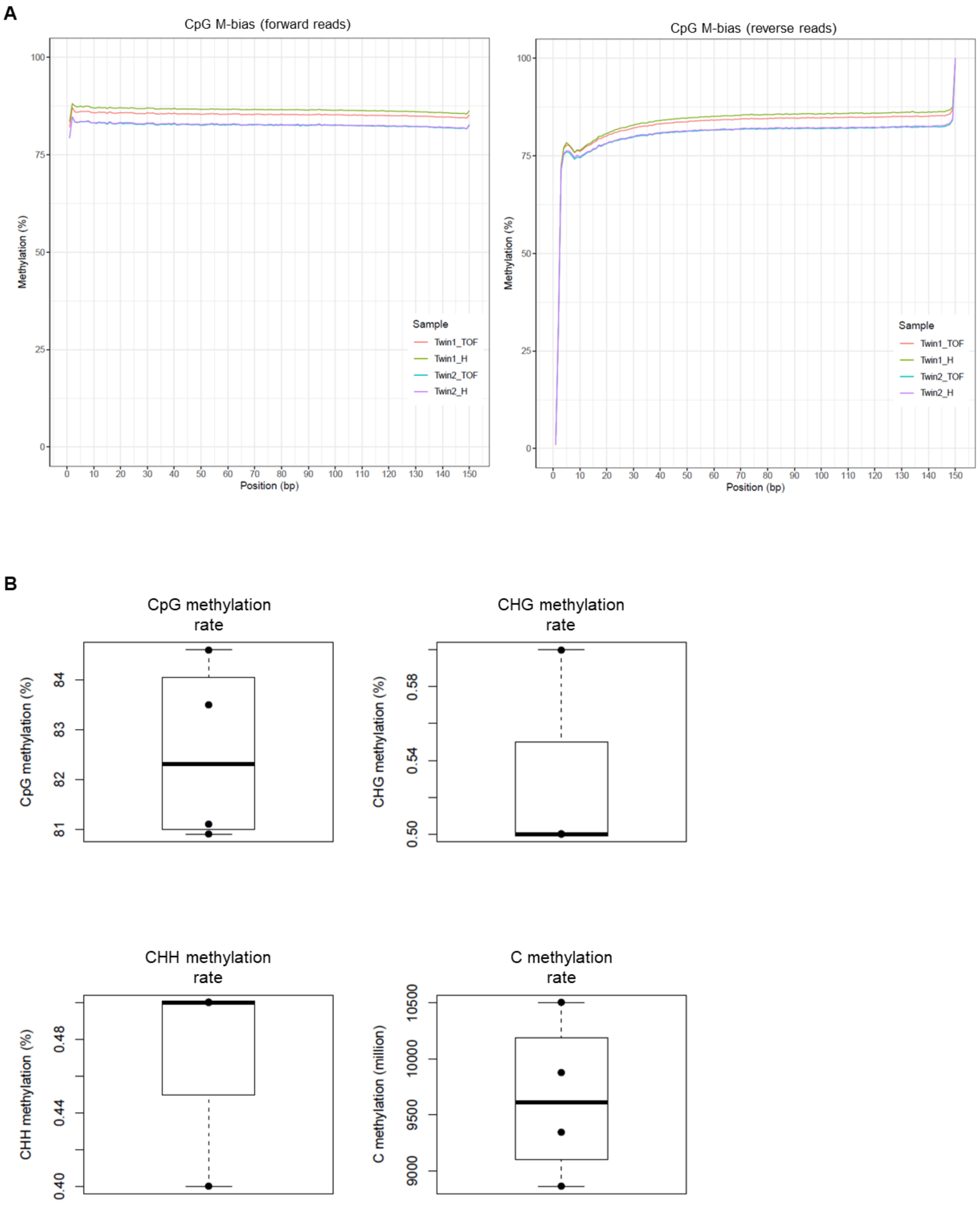

Figure S5. Methylation bias and rates. (**A**) CpG methylation bias (M-bias) over forward and reverse reads over all bases. (**B**) Methylation rates over CpGs, CHGs, CHHs and Cs. Note that 'H' denotes for A, T or C.

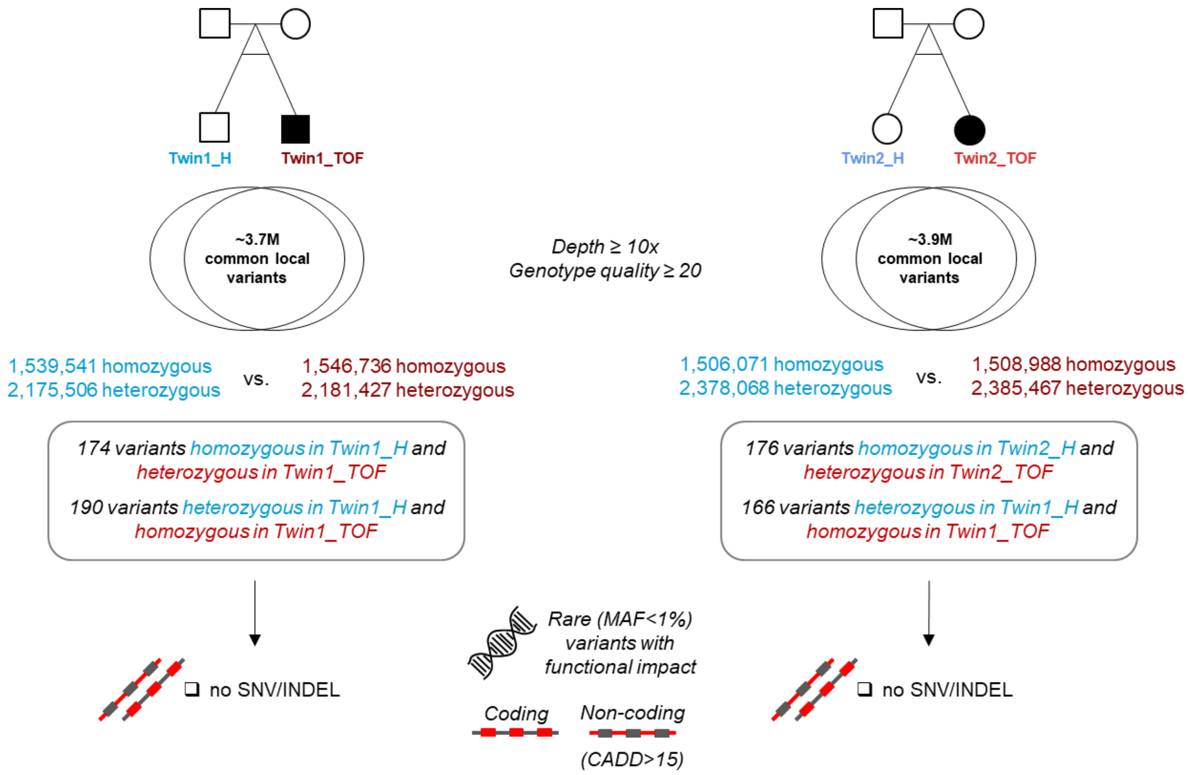

Figure S6. Filtering of possible disease-relevant local variations identified in affected TOF twins based on zygosity differences between healthy and affected sibling. CADD, combined annotation dependent depletion; H, healthy; INDEL, insertion and deletion; M, million; MAF, minor allele frequency; SNV, single nucleotide variation; TOF, Tetralogy of Fallot.

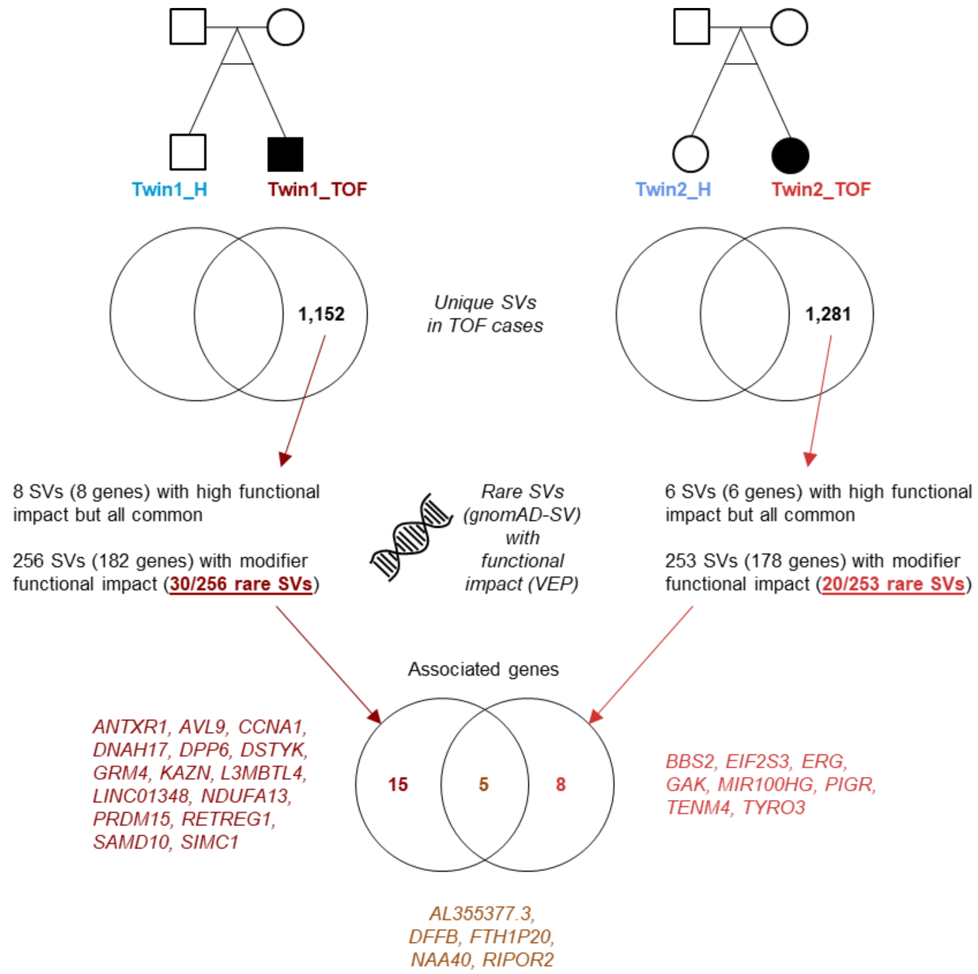

Figure S7. Filtering of possible disease-relevant structural variations identified in affected TOF twins using whole genome sequencing. H, healthy; TOF, Tetralogy of Fallot; SV, structural variation; VEP, Ensembl variant effect predictor.

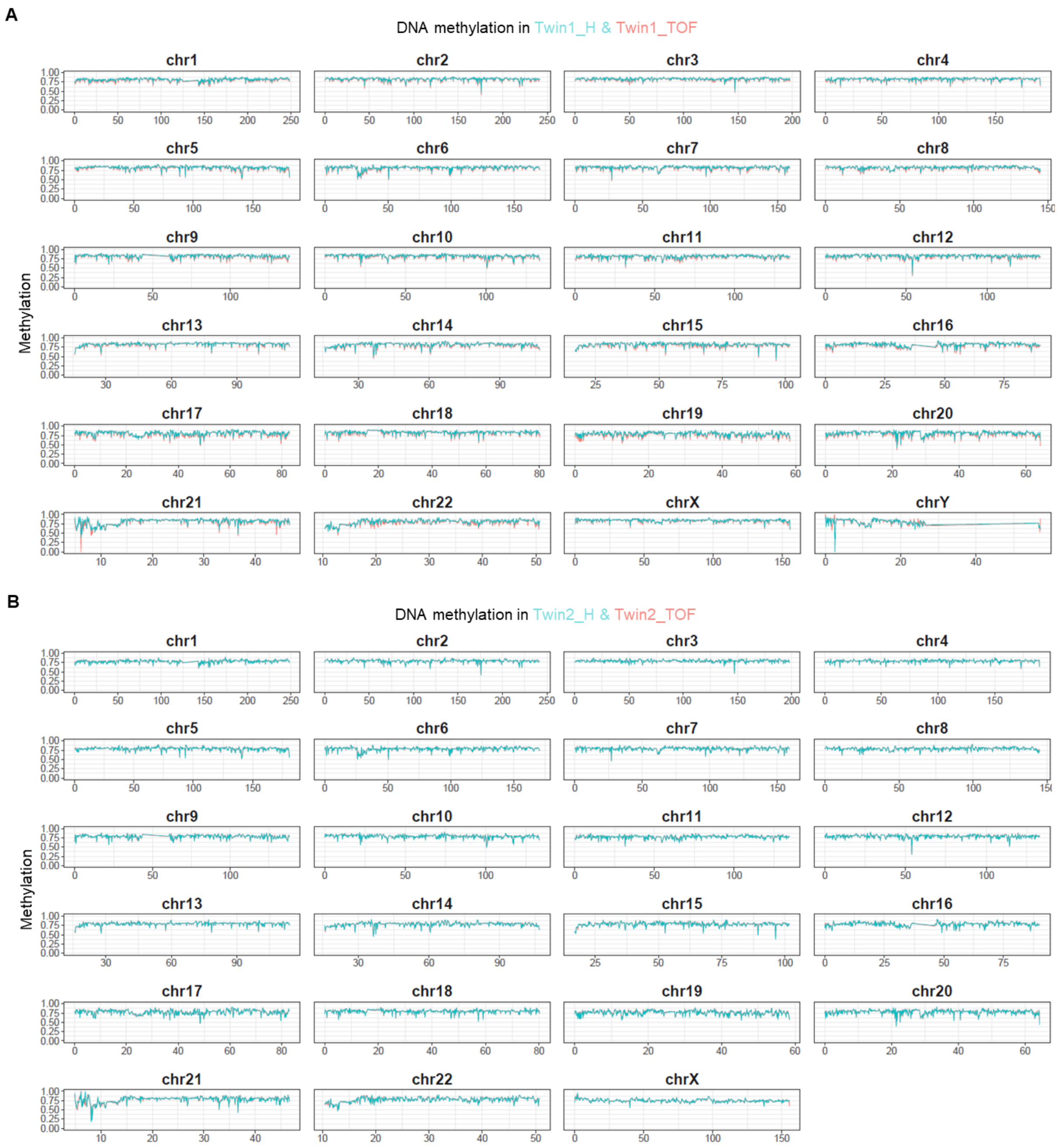

Figure S8. Global DNA methylation levels of twins. Line plots show the DNA methylation along the chromosomal length (GRCh38.p13/hg38). (**A**) Methylation levels in Twin1_H and Twin1_TOF. (**B**) Methylation levels in Twin2_H and Twin2_TOF. H, healthy; TOF, Tetralogy of Fallot.

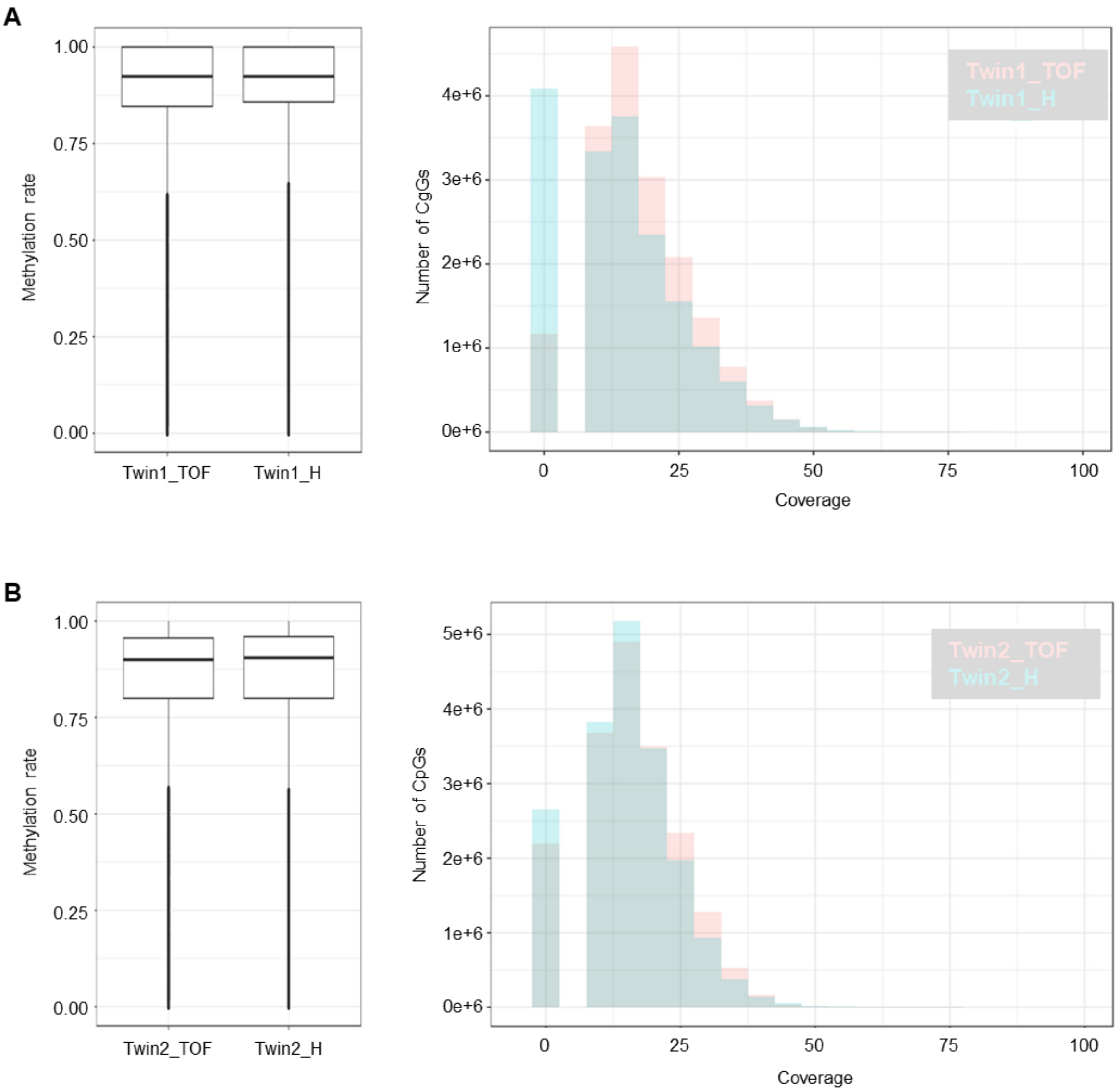

Figure S9. DNA methylation rate and coverage of CpGs. (**A**) Methylation and coverage in Twin1_H and Twin1_TOF. (**B**) Methylation and coverage in Twin2_H and Twin2_TOF. H, healthy; TOF, Tetralogy of Fallot.

Sample		Dups (%)	GC (%)	Total reads (million)	Mean base quality (Pred)
Twin1_H	Twin1_H_TD180611543_HNCVWCCXY_L4_1	11.70%	40%	78.5	38.6
	Twin1_H_TD180611543_HNCVWCCXY_L4_2	10.50%	41%	78.5	37.2
	Twin1_H_TD180611543_HNCVWCCXY_L5_1	11.30%	40%	77.5	38.6
	Twin1_H_TD180611543_HNCVWCCXY_L5_2	10.70%	41%	77.5	37.2
	Twin1_H_TD180611543_HNCVWCCXY_L6_1	10.30%	40%	78.3	38.7
	Twin1_H_TD180611543_HNCVWCCXY_L6_2	10.30%	41%	78.3	37.3
	Twin1_H_TD180611543_HNCVWCCXY_L7_1	10.90%	40%	78.0	38.6
	Twin1_H_TD180611543_HNCVWCCXY_L7_2	10.30%	41%	78.0	37.2
Twin1_TOF	Twin1_TOF_TD180611542_HNCVWCCXY_L4_1	10.70%	40%	79.6	38.6
	Twin1_TOF_TD180611542_HNCVWCCXY_L4_2	9.60%	41%	79.6	37.0
	Twin1_TOF_TD180611542_HNCVWCCXY_L5_1	10.10%	40%	79.0	38.6
	Twin1_TOF_TD180611542_HNCVWCCXY_L5_2	9.60%	41%	79.0	37.0
	Twin1_TOF_TD180611542_HNCVWCCXY_L6_1	9.30%	40%	79.8	38.6
	Twin1_TOF_TD180611542_HNCVWCCXY_L6_2	9.10%	41%	79.8	37.1
	Twin1_TOF_TD180611542_HNCVWCCXY_L7_1	9.90%	40%	79.4	38.6
	Twin1_TOF_TD180611542_HNCVWCCXY_L7_2	9.20%	41%	79.4	37.0
Twin2_H	Twin2_H_TD180611545_HNCVWCCXY_L4_1	11.30%	40%	85.3	38.6
	Twin2_H_TD180611545_HNCVWCCXY_L4_2	10.20%	41%	85.3	37.2
	Twin2_H_TD180611545_HNCVWCCXY_L5_1	10.80%	40%	84.8	38.6
	Twin2_H_TD180611545_HNCVWCCXY_L5_2	10.20%	41%	84.8	37.2
	Twin2_H_TD180611545_HNCVWCCXY_L6_1	10.00%	40%	85.6	38.6
	Twin2_H_TD180611545_HNCVWCCXY_L6_2	9.90%	41%	85.6	37.3
	Twin2_H_TD180611545_HNCVWCCXY_L7_1	10.70%	40%	85.3	38.6
	Twin2_H_TD180611545_HNCVWCCXY_L7_2	10.00%	41%	85.3	37.2
Twin2_TOF	Twin2_TOF_TD180611544_HNCVWCCXY_L4_1	11.70%	40%	89.7	38.7
	Twin2_TOF_TD180611544_HNCVWCCXY_L4_2	10.40%	40%	89.7	37.3
	Twin2_TOF_TD180611544_HNCVWCCXY_L5_1	11.40%	40%	89.1	38.6
	Twin2_TOF_TD180611544_HNCVWCCXY_L5_2	10.70%	40%	89.1	37.3
	Twin2_TOF_TD180611544_HNCVWCCXY_L6_1	10.70%	40%	90.0	38.7
	Twin2_TOF_TD180611544_HNCVWCCXY_L6_2	10.40%	40%	90.0	37.4
	Twin2_TOF_TD180611544_HNCVWCCXY_L7_1	11.20%	40%	89.7	38.6
	Twin2_TOF_TD180611544_HNCVWCCXY_L7_2	10.40%	40%	89.7	37.3
	average	*10.42%*	*40.4%*	*83.1*	*37.91*

Table S1. Overview of sequencing reads obtained from whole genome sequencing. Paired-end Illumina sequencing (2x150 bp) was performed. For each sample, 'L' denotes the sequencing lane and '*_1'/'*_2' represents the forward and reverse reads, respectively.

	Twin1_H	Twin1_TOF	Twin2_H	Twin2_TOF
**Total reads**	623696038 (100%)	634620440 (100%)	681201156 (100%)	715910380 (100%)
**Duplicate**	77812477 (12.5%)	71476214 (11.3%)	85443090 (12.5%)	92229860 (12.9%)
**Mapped reads**	616017435 (98.8%)	627147782 (98.82%)	672959701 (98.8%)	707412206 (98.8%)
**PE mapped**	612513294 (98.2%)	623523880 (98.3%)	669238718 (98.2%)	703618084 (98.2%)
**SE mapped**	3504141 (0.6%)	3623902 (0.6%)	3720983 (0.5%)	3794122 (0.5%)
**Average sequencing depth**	28.25	28.76	30.87	32.45
**Coverage**	99.82%	99.82%	99.21%	99.22%
**Coverage ≥4X**	98.79%	98.79%	98.20%	98.25%
**Coverage ≥10X**	96.28%	96.28%	96.31%	96.48

Table S2. Statistics of read mapping, coverage and read depth in each sample obtained from whole genome sequencing. PE, paired-end; SE, single-end.

Sample		Dups (%)	GC (%)	Total reads (million)	Mean base quality (Pred)
Twin1_TOF	Sp333_L1_1	10.7%	21%	60.7	38.61
	Sp333_L1_2	7.2%	21%	60.7	35.96
	Sp333_L2_1	10.8%	21%	60.9	38.59
	Sp333_L2_2	8.8%	21%	60.9	35.42
	Sp333_L3_1	11.1%	21%	61.5	38.62
	Sp333_L3_2	10.9%	21%	61.5	35.93
	Sp333_L4_1	11.4%	21%	60.7	38.59
	Sp333_L4_2	10.8%	21%	60.7	35.84
	Sp333_L5_1	11.4%	21%	60.8	38.57
	Sp333_L5_2	10.7%	21%	60.8	35.88
	Sp333_L6_1	11.6%	21%	60.8	38.59
	Sp333_L6_2	10.6%	21%	60.8	35.79
Twin1_H	Sp334_L1_1	9.5%	21%	52.8	38.61
	Sp334_L1_2	6.9%	21%	52.8	35.28
	Sp334_L2_1	9.6%	21%	52.9	38.60
	Sp334_L2_2	7.9%	21%	52.9	34.74
	Sp334_L3_1	9.9%	21%	53.1	38.62
	Sp334_L3_2	9.3%	21%	53.1	35.21
	Sp334_L4_1	10.1%	21%	52.9	38.60
	Sp334_L4_2	9.4%	21%	52.9	35.11
	Sp334_L5_1	10.2%	21%	53.0	38.58
	Sp334_L5_2	9.4%	21%	53.0	35.16
	Sp334_L6_1	10.2%	21%	52.9	38.59
	Sp334_L6_2	9.2%	21%	52.9	35.06
Twin2_TOF	Sp335_L1_1	9.9%	21%	58.1	38.57
	Sp335_L1_2	6.7%	21%	58.1	35.33
	Sp335_L2_1	10.3%	21%	58.4	38.56
	Sp335_L2_2	8.4%	21%	58.4	34.87
	Sp335_L3_1	10.5%	21%	58.5	38.58
	Sp335_L3_2	10.1%	21%	58.5	35.36
	Sp335_L4_1	10.8%	21%	58.1	38.56
	Sp335_L4_2	10.2%	21%	58.1	35.26
	Sp335_L5_1	10.8%	21%	58.2	38.53
	Sp335_L5_2	10.1%	21%	58.2	35.30
	Sp335_L6_1	10.9%	21%	58.2	38.55
	Sp335_L6_2	10.0%	21%	58.2	35.21
Twin2_H	Sp336_L1_1	10.6%	21%	57.4	38.53
	Sp336_L1_2	7.3%	22%	57.4	35.32
	Sp336_L2_1	10.7%	21%	57.5	38.52
	Sp336_L2_2	8.7%	22%	57.5	34.81
	Sp336_L3_1	10.8%	21%	57.7	38.54
	Sp336_L3_2	10.3%	22%	57.7	35.30
	Sp336_L4_1	11.0%	21%	57.4	38.52
	Sp336_L4_2	10.3%	22%	57.4	35.20
	Sp336_L5_1	11.2%	21%	57.5	38.50
	Sp336_L5_2	10.3%	22%	57.5	35.25
	Sp336_L6_1	11.3%	21%	57.4	38.51
	Sp336_L6_2	10.2%	22%	57.4	35.15
	average	*10.0%*	*21.1%*	*57.4*	*36.95*

Table S3. Overview of sequencing reads obtained from whole genome bisulfite sequencing. Paired-end Illumina sequencing (2x150 bp) was performed. For each sample, 'L' denotes the sequencing lane and '*_1'/'*_2' represents the forward and reverse reads, respectively.

Sample		Total reads	Aligned reads	Unaligned reads	Ambiguously aligned reads	Duplicates (removed)	Unique reads (remaining)
Twin1_TOF	Sp333_RDM00799_L1	60713900	47385992	11020584	2307323	5189315	42196677
	Sp333_RDM00799_L2	60893917	46199044	12445190	2249682	5134995	41064049
	Sp333_RDM00799_L3	61492618	47941470	11220346	2330802	5487141	42454329
	Sp333_RDM00799_L4	60709131	47151752	11261855	2295523	5388325	41763427
	Sp333_RDM00799_L5	60734267	47279615	11151329	2303322	5500015	41779600
	Sp333_RDM00799_L6	60772843	47201470	11270561	2300811	5579960	41621510
Twin1_H	Sp334_RDM00800_L1	52811578	42765055	8112712	1933811	3788216	38976839
	Sp334_RDM00800_L2	52869973	41547389	9448694	1873888	3745131	37802258
	Sp334_RDM00800_L3	53073278	42862058	8277457	1933762	3975946	38886112
	Sp334_RDM00800_L4	52907527	42575157	8410260	1922110	3951599	38623558
	Sp334_RDM00800_L5	52970756	42740812	8298848	1931094	4042902	38697910
	Sp334_RDM00800_L6	52880739	42553677	8406457	1920605	4086371	38467306
Twin2_TOF	Sp335_RDM00801_L1	58057558	43962495	11948026	2147033	4600494	39362001
	Sp335_RDM00801_L2	58331772	42931982	13304995	2094791	4569984	38361998
	Sp335_RDM00801_L3	58434542	44208796	12070948	2154794	4838393	39370403
	Sp335_RDM00801_L4	58102451	43793880	12175556	2133013	4789273	39004607
	Sp335_RDM00801_L5	58145454	43929444	12074280	2141726	4889574	39039870
	Sp335_RDM00801_L6	58218510	43881767	12194631	2142107	4963750	38918017
Twin2_H	Sp336_RDM00802_L1	57337526	38671981	16656058	2009485	3765512	34906469
	Sp336_RDM00802_L2	57461325	37640382	17867931	1953011	3723665	33916717
	Sp336_RDM00802_L3	57656206	38835755	16807423	2013024	3947861	34887894
	Sp336_RDM00802_L4	57403215	38509393	16895107	1998711	3912006	34597387
	Sp336_RDM00802_L5	57474641	38653792	16814326	2006521	4003857	34649935
	Sp336_RDM00802_L6	57411843	38510060	16898712	2003071	4044545	34465515

Table S4. Mapping result of reads obtained from whole genome bisulfite sequencing. For each sample, 'L' denotes the sequencing lane.

	Twin1_H	Twin1_TOF	Twin2_H	Twin2_TOF
**Mean depth**	24.0	22.1	22.4	19.8
**SD depth**	37.31	37.57	36.59	43.77
**Mean mapping quality (Pred)**	36.01	35.83	35.62	35.43
**Mean insert size (bp)**	256.24	255.93	251.37	255.33
**SD insert size (bp)**	54.78	56.80	55.08	56.77
**GC (%)**	20.9	20.6	21.5	22.2
**Methylated CpG (%)**	83.5	84.6	80.9	81.1
**Methylated CHG (%)**	0.5	0.6	0.5	0.5
**Methylated CHH (%)**	0.4	0.5	0.5	0.5
**Cs (million)**	10504	9347	9875	8860
**Dups (%)**	12.1	10.0	11.5	11.1
**Deduplicated reads (million)**	249.1	229.5	232.4	205.2
**Duplicated reads (million)**	34.1	25.5	30.3	25.6

Table S5. Read statistics after mapping and deduplication as well as methylation rates over CpGs, CHGs and CHHs in each sample obtained from whole genome bisulfite sequencing.

	Chr	Start	End	Copy number	Type	Associated gene(s)
Twin1_TOF	4	68550000	68600000	4	gain	UGT2B17
	5	46450000	46500000	3	gain	-
	7	38250000	38350000	3	gain	TRGJ2, TRGJP2, TRGC1, TRGJ1, TRGJP, TARP
	9	60700000	60750000	3	gain	-
	10	39650000	39700000	3	gain	-
	13	86200000	86250000	3	gain	-
	18	20850000	20900000	1	loss	-
	19	54850000	54900000	3	gain	KIR3DL2, FCAR
	21	5450000	5500000	3	gain	-
	21	5800000	5850000	3	gain	-
	21	7150000	7200000	1	loss	-
Twin2_TOF	1	228550000	228600000	3	gain	RNA5SP19
	5	46450000	46500000	3	gain	-
	14	41150000	41200000	1	loss	-
	18	20850000	20900000	1	loss	-

Table S6. Copy number variations identified in Twin1_TOF and Twin2_TOF based on whole genome sequencing data. Positions based human reference genome (GRCh38.p13/hg38).

Chr	Pos	ID	REF	ALT	Assoicated gene	GT:FT:GQ:PL:PR(:SR)
chr1	3868664	MantaINS:117:0:0:0:2:0	A	ACCACACCAGGCCACACCACACCACACCACACCACGCCACACCACAC CAGGCCACACCACACCACG	DFFB	1/1:MinGQ:5:65,7,0:0,0:0,6
chr1	14867953	MantaINS:523:0:0:0:0:0	A	AGCATTGCATAGGCATGGCATCACATACACGAGCATTGCATAGGCACG GCACAGCATATGCGG	KAZN	0/1:PASS:55:105,0,178:0,0:16,3
chr1	16725226	MantaBND:616:0:1:0:0:0:0	A	[chr1:234776356[A		0/1:MinGQ:12:62,0,821:55,17
chr1	205209478	MantaBND:6064:0:1:0:0:0:1	C	CACAAC]chr1:205209646]	DSTYK	0/1:PASS:203:339,0,200:9,2:18,7
chr1	205209646	MantaBND:6064:0:1:0:0:0:0	A	AGTTGT]chr1:205209478]	DSTYK	0/1:PASS:203:339,0,200:9,2:18,7
chr1	235078305	MantaDUP:TANDEM:7137:0:1:0:1:0	G	<DUP:TANDEM>	LINC01348	0/1:PASS:500:718,0,497:19,4:27,24
chr11	13745959	MantaDEL:58994:0:0:0:0:0	CTTCAATACCTGTGAAAAGAAGGGAGTGAAAGCTGGATTGGGCAAAGA AAGAAATCAAGCTGTGTTGCAGGCCTGATAAATTCTCAGTCCCACGGTG GTG	CCACGGTGGGGAGCTCAGGAGTCA		1/1:PASS:50:892,53,0:0,0:0,18
chr11	63931436	MantaDEL:60668:0:1:0:0:0	A	<DEL>	NAA40	0/1:PASS:348:576,0,345:10,6:25,12
chr13	36433387	MantaBND:68499:0:1:0:0:0:0	T	]chr22:17720154]T	CCNA1	0/1:PASS:51:101,0,201:9,0:16,3
chr17	78550343	MantaINS:82409:0:0:0:0:0	T	TGCCCAGCACTGAAACTGAGGCAGGGCTGGTAGGGGCTGAACTGTGCC CCCCAAATCCATGTGTAGGG	DNAH17	0/1:PASS:147:197,0,172:0,0:11,5
chr18	6316029	MantaDEL:82892:0:0:0:0:0	TCACTCTCACGGAGTCCATGCCACTCCCCTGCCACCCCCATCGGAGCTA GTGCTGGTGCCTACTGCTGGGAGACATAAGGAAACAGTTACGTC	TT	L3MBTL4	0/1:PASS:222:517,0,219:4,4:20,12
chr19	19521618	MantaBND:86372:1:4:0:0:0:1	T	T]chr22:15630722]	NDUFA13	0/1:PASS:137:187,0,999:23,1:68,10
chr2	69096281	MantaDEL:10188:0:0:0:0:0	GAAGGAAGGGAGGAAGGGAGGAAGGGAGGAAGGGAGGAAGGGAGG AAGGGAGGAAGGGAGGAAGGG	GC	ANTXR1	1/1:PASS:17:219,20,0:0,0:0,9
chr2	180873533	MantaINS:13464:0:0:0:0:1	C	CGGCCGGCCGGGGTGGGGGACGAGCGCCGGGTTCCGTCCAAGCCCT GTTGAAGCAG	FTH1P20	1/1:MinGQ:7:150,9,0:0,0:0,3
chr20	24836274	MantaINS:88780:0:0:0:2:0	A	<INS>		1/1:PASS:41:670,44,0:0,3:0,14
chr20	63970889	MantaDEL:90162:0:0:0:0:0	TCTTCCTTCCCTTACCTTCCTCCCTCCCTTCTTCCTTCCTTTCCTTCCTTCTTC	T	SAMD10	0/1:MinGQ:10:399,0,6:2,0:13,11
chr21	38218604	MantaDEL:91274:0:0:0:0:0	TCACCTGGGCTGTTTTTTTTTTTTTTTTTTTTTTTTTTTTTTTTTTTTTTTTTTTTTTTT TTTTTTGAGACGGAGTCTCGCTCTGTCGCCCAGGCTGGAGTGCAGTGGC GGGATCTCGGCTCACTGCAAGCTCCGCCTCCCGGGTTCACGCCATTCTC CTGCCTCAGCCTCCCAAGTAGCTGGGACTACAGGCGCCCGCCACTACG CCCGGCTAATTTTTTTTGTATTTTTAGTAGAGACGGGGTTTCACCGTTTTAG CCGGGATGGTCTCGATCTCCTGACCTCGTGATCCGCCCGCCTCGGCCTC CCAAAGTGCTGGGATTACAGGCGTGAGCCACCGCGCCCGGC	T		1/1:PASS:15:140,18,0:0,6:0,4
chr21	41835843	MantaINS:91423:0:0:0:0:0	CT	CCTCACCTGCTCTCATCCCTCCTGGGACTCTCTTCTCAGGGACGCTTGGA CACCCACAGCCC	PRDM15	0/1:PASS:135:278,0,132:0,0:9,7
chr5	13598560	MantaINS:29091:0:0:0:0:0	T	TATGTATGATGATACATCATGCATGATACATGTATGATGATACATCGTGC ATGATAC		1/1:PASS:30:495,33,0:0,0:0,11
chr5	16525104	MantaDEL:29178:0:0:0:0:0	CCCTGCAGGTGGATGTGCGTGGGGGACACTGTGATGACCTGTGTCCTCC GGCT	C	RETREG1	0/1:PASS:67:224,0,64:1,0:8,6
chr5	16525277	MantaDEL:29178:0:0:0:0:1	CGCGGGGGGACACTGTGCTGATTTGCGTCCTCCAGCCCCAACAGGTGG ATGTGCG	CA	RETREG1	0/1:PASS:154:504,0,151:0,0:14,12
chr5	176306931	MantaBND:7212:0:1:0:0:0:0	A	A]chr1:236374908]	SIMC1	0/1:PASS:17:67,0,499:31,3:11,2
chr6	7717134	MantaDEL:34605:0:0:0:0:0	TAAAGAATGGCTAAAGAGGCCGGGCGCGGTGGCTCACGCCTGTAATCC CAGCACTTTGGGAGGCCGAGGCGGGCGGATCACGAGGTCAGGAGATC GAGACCATTCTGGCTAACACGGTGAAACCCCGTCTCTACTAAAAATACA AAAAATTAGCCGGGCGTGGTAGCGGGCGCCTGTAGTCCCAGCTACTCG GGAGGCTGAGGCAGGAGAATGGCGTGAACCCGGGAGGCGGAGCTTGC AGTGAGCCGAGATCGCGCCACTGCACTCCAGCCTGGGCGACAGAGCG AGACTCCGTCTCAAAAAAAAAAAAAAAAAAAAAAAAAAAAAAAAAAAAA AA	T		1/1:MinGQ:8:115,10,0:0,4:0,2
chr6	24811710	MantaDEL:35350:0:1:0:0:0	G	<DEL>	RIPOR2	0/1:MinGQ:4:213,2,0:2,11
chr6	34074475	MantaDEL:35533:0:0:0:0:0	ACCAGGAGGCTGCTGGGCAGCGCAGGCAGTGTGGCTCAGAGCTGAGC CCTGCCGTCCCCTGCCAGGAGAGGAGGCTGCTGGGCGGCGCAGGCAG TGTGGCTCAGAGCTGAGCCCTGCCGTCCCCTG	A	GRM4	0/1:PASS:190:476,0,187:7,2:14,12
chr7	32531415	MantaINS:41174:0:0:0:0:0	G	GAACATGTATATGAACATGTATATGTTAATGAACATGAATATACATGTTCA TATACATGTTCATT	AVL9	1/1:PASS:15:279,18,0:0,0:2,7
chr7	154794795	MantaBND:45134:0:1:0:0:0:1	C	C[chrX:24347205[	DPP6	0/1:PASS:112:198,0,109:9,12
chr7	154794811	MantaINS:45134:0:0:0:0:0	T	TGGGCCCCCCCACCCCCACCCCCACCCCCACCCCCACCCCACGCTGCT CCATCA	DPP6	1/1:MinGQ:12:248,15,0:0,0:0,6
chr9	35018979	MantaINS:50857:0:0:0:0:0	T	TGTGTTGTTCCTCTCATGTGTCCATGTGTTTTCATTGTTCAGCTCCCACTTA TAAGTGAGAACAC	AL355377.3	1/1:PASS:36:627,39,0:0,0:0,13
chrX	45434811	MantaDEL:93747:0:0:0:0:0	TTTTTTTAACGGTAAGTCTGCTAGTGACAGATCCCTTTAATCTTCCTTTGTC TGAGAATATC	T		1/1:PASS:21:384,24,0:0,0:0,8

Table S7. Structural variations uniquely identified in Twin1_TOF based on whole genome sequencing data. Positions based human reference genome (GRCh38.p13/hg38).

Chr	Pos	ID	REF	ALT	Assoicated gene	GT:FT:GQ:PL:PR(:SR)
chr1	3868664	MantaINS:126:0:0:0:0:0	A	ACCACCCCAGGCCACGCCACACCAAGCCACACCACACCAGGCCACACCACACCAGGCCACACCACACCATACCACACCACACCAGGCCACACCACACCAGGCCACACCACACCACGCCACACCACACCACGCCACACCACACCAGG	DFFB	1/1:PASS:25:387,28,0:0,1:0,16
chr1	16725255	MantaBND:687:0:1:0:0:0:0	C	[chr1:234776370[C		0/1:PASS:297:347,0,488:36,29
chr1	206923954	MantaDUP:TANDEM:6517:0:1:0:0:0	T	<DUP:TANDEM>	PIGR	0/1:PASS:30:80,0,593:19,4:34,5
chr11	63931436	MantaDEL:64286:0:1:0:0:0	A	<DEL>	NAA40	0/1:PASS:431:481,0,568:25,10:35,8
chr11	78805275	MantaINS:64833:0:0:0:0:0	T	TCTCCCCACCCTCCACACCCCACCCCTCATTCTTCCCCACCCCCACTCCTCCTCCACCCACCCTTC	TENM4	1/1:PASS:33:557,36,0:0,0:0,12
chr11	122084984	MantaDEL:66360:0:0:0:0:0	AAAAATATATATATATATATATATATAATATACATGATATATCATGTATAT	ATATA	MIR100HG	0/1:PASS:20:227,0,17:1,0:3,5
chr15	41559198	MantaBND:72779:0:2:0:0:0:0	C	[chr13:43495691[GC	TYRO3	0/1:PASS:195:245,0,488:10,7:25,5
chr16	56518771	MantaINS:82820:0:0:0:0:0	A	AGCATTTTGGGCATGCCTCCGTCAAATACACAGGGCTCTGATTTGCCTTGTCTG	BBS2	0/1:PASS:174:224,0,305:1,0:20,7
chr2	180873533	MantaINS:14204:0:0:0:0:1	C	CGGCCGGCCGGGGTGGGGGACGAGCGCCGGGTTCCGTCCAAGCCCTGTTGAAGCAG	FTH1P20	1/1:MinGQ:5:98,6,0:0,0:0,2
chr20	6665221	MantaBND:79053:1:2:0:0:0:0	T	T[chr15:44985517[		0/1:PASS:62:112,0,343:24,12
chr21	38218604	MantaDEL:96321:0:0:0:0:0	TCACCTGGGCTGTTTTTTTTTTTTTTTTTTTTTTTTTTTTTTTTTTTTTTTTTTTTTTTTTTTTTTTTTTTTTTGAGACGGAGTCTCGCTCTGTCGCCCAGGCTGGAGTGCAGTGGCGGGATCTCGGCTCACTGCAAGCTCCGCCTCCCGGGTTCACGCCATTCTCCTGCCTCAGCCTCCCAAGTAGCTGGGACTACAGGCGCCCGCCACTACGCCCGGCTAATTTTTTTTTGTATTTTTAGTAGAGACGGGGTTTCACCGTTTTAGCCGGGATGGTCTCGATCTCCTGACCTCGTGATCCGCCCGCCTCGGCCTCCCAAAGTGCTGGGATTACAGGCGTGAGCCACCGCGCCCGGC	T		1/1:PASS:16:178,19,0:0,6:0,4
chr21	38429549	MantaDEL:96299:0:0:0:0:1	GTATATGTACATGTATACACATGTACATATATACATATGTGTATATGTACA	G	ERG	1/1:MinGQ:12:248,15,0:0,0:0,5
chr4	856468	MantaDEL:23710:0:0:0:0:1	GCTGCTCACCACCACAGCTGCTCACACCTACTCACCACCACAGCTGCTCACAC	G	GAK	0/1:PASS:147:198,0,149:3,0:14,5
chr6	24811647	MantaDEL:37456:0:1:0:0:0	T	<DEL>	RIPOR2	1/1:PASS:21:237,24,0:0,6:0,2
chr6	87869372	MantaINS:39405:0:0:0:0:0	G	GTCTTATGGGTGATTATGTCATGACACAAAGTGTTCTCCTGATCTTATCTC		1/1:PASS:32:558,35,0:0,0:0,13
chr7	41574808	MantaDEL:43857:0:0:0:0:0	GAAATACTTTAAGAAACCCTGTGTTAAAACCCTGTGTTAAAACTCTGTATTTAAAACAGTT	G		0/1:PASS:147:197,0,189:4,0:16,5
chr7	58064116	MantaBND:44429:0:1:0:1:0:1	A	[chr16:36124088[A		0/1:PASS:304:376,0,301:13,6:19,43
chr7	151312872	MantaBND:47612:1:2:0:0:0:1	T	[chr7:151315024[T		0/1:PASS:20:70,0,260:18,8
chr9	35018979	MantaINS:54122:0:0:0:0:0	T	TGTGTTGTTCCTCTCATGTGTCCATGTGTTTTCATTGTTCAGCTCCCACTTATAAGTGAGAACAC	AL355377.3	1/1:PASS:18:268,21,0:0,0:0,8
chrX	24083251	MantaDEL:98813:0:0:0:0:0	TAAAAAGACTTAGCAGGGCCGGGCGCGGTGGCTCACGCCTGTAATCCCAGCACTTTGGGAGGCCGAGGCGGGCGGATCACGAGGTCAGGAGATCGAGACCATTCTGGCTAACACGGTGAAACCCCGTCTCTACTAAAAATACAAAAAATTAGCCGGGCGTGGTAGCGGGCGCCTGTAGTCCCAGCTACTCGGGAGGCTGAGGCAGGAGAATGGCGTGAACCCGGGAGGCGGAGCTTGCAGTGAGCCGAGATCGCGCCACTGCACTCCAGCCTGGGCGACAGAGCGAGACTCCGTCTCAAAAACAAAAAAAAAAAAA	T	EIF2S3	1/1:MinGQ:13:254,16,0:0,3:0,5

Table S8. Structural variations uniquely identified in Twin2_TOF based on whole genome sequencing data. Positions based human reference genome (GRCh38.p13/hg38).

	Gene	Overlap	Tissue (RV)		iPSC-derived CMs (day 15)		iPSC-derived CMs (day 60)	
			NH (n=4)	TOF (n=18)	Healthy (n=2; 2-3 clones each)	TOF (n=1, 2 clones)	Healthy (n=2; 3 clones each)	TOF (n=1, 3 clones)
Twin1_TOF & Twin2_TOF	*AL355377.3*		0.0	0.0	0.0	0.0	0.0	0.0
	DFFB	*1,4*	2.6	3.9	7.3	8.7	4.4	2.7
	FTH1P20		0.0	0.0	4.4	3.7	6.4	6.0
	NAA40	*3*	6.0	9.2	14.2	20.4	14.5	13.7
	RIPOR2		0.0	0.0	0.0	0.0	0.0	0.0
Twin1_TOF	*ANTXR1*	*2,3*	4.5	9.9	24.7	29.5	64.7	24.9
	AVL9		2.1	1.9	6.2	6.1	4.3	3.0
	CCNA1		0.0	0.0	0.6	0.1	0.4	0.4
	DNAH17		0.2	0.2	0.5	0.4	0.2	0.3
	DPP6		0.1	0.1	0.4	0.4	0.1	0.3
	DSTYK		2.0	2.5	4.4	3.7	4.8	2.7
	GRM4	*3*	0.1	0.2	0.4	0.1	0.0	0.1
	KAZN	*3*	0.7	0.5	6.4	6.1	4.1	1.5
	L3MBTL4	*2,3,4,6*	0.2	1.2	4.5	2.6	4.5	3.4
	LINC01348		0.0	0.0	0.0	0.0	0.0	0.0
	NDUFA13	*1*	1836.7	1749.8	114.7	163.8	123.0	203.7
	PRDM15		1.3	1.0	2.5	3.1	2.1	1.5
	RETREG1		0.0	0.0	0.0	0.0	0.0	0.0
	SAMD10	*4*	2.4	1.9	2.0	1.6	1.2	1.6
	SIMC1	*4*	0.5	0.7	12.1	10.2	6.6	3.8
Twin2_TOF	*BBS2*	*1,3*	7.1	8.6	54.6	45.0	34.2	34.2
	EIF2S3	*1*	13.0	18.3	184.1	110.4	121.5	52.3
	ERG		3.4	3.8	0.6	0.3	3.0	0.2
	GAK	*4*	7.0	7.5	17.1	20.1	12.3	10.6
	MIR100HG		3.7	7.0	2.0	1.6	2.5	0.6
	PIGR		0.7	0.8	0.1	0.0	0.0	0.0
	TENM4	*3*	0.1	0.1	8.8	7.2	3.5	1.7
	TYRO3		1.1	1.1	15.3	26.0	8.1	4.3

Table S9. Candidate genes with structural variations in Twin1_TOF and/or Twin2_TOF. Expression is given in RPKM (tissue; Grunert *et al.* 2014) and TPM (CMs; Grunert *et al.* 2020) values, respectively. Overlap with list of cardiovascular-associated genes is indicated by '1' and overlaps with CHD-related datasets are indicated by '2' (expression), '3' (methylation), '4' (CNV), '5' (CHD gene), and '6' (miRtarget). CHD, congenital heart disease; CNV, copy number variation; CMs, cardiomyocytes; H, healthy; iPSC, induced pluripotent stem cell; NH, normal heart; RPKM, reads per kilo base per million mapped reads; RV, right ventricle; TOF, Tetralogy of Fallot; TPM, transcript per million.
